# Outcomes of home design to support healthy cognitive ageing: modified e-Delphi exercise with older people and housing-related professionals

**DOI:** 10.1186/s12877-024-05085-z

**Published:** 2024-06-24

**Authors:** Alison Bowes, Lisa Davison, Alison Dawson, Catherine Pemble

**Affiliations:** Faculty of Social Sciences, University of Stirling, Stirling, FK9 4LA UK

**Keywords:** E-Delphi, Cognitive ageing, Dementia, Healthy ageing, Home design, Older people, Professionals

## Abstract

**Background:**

There is emerging agreement that living in a home designed to support healthy cognitive ageing can enable people to live better with dementia and cognitive change. However, existing literature has used a variety of outcome measures that have infrequently been informed by the perspectives of older people or of professional in design and supply of housing. The DesHCA (Designing Homes for Healthy Cognitive Ageing) study aimed to identify outcomes that were meaningful for these groups and to understand their content and meanings.

**Methods:**

A presurvey of older people and housing professionals (*n* = 62) identified potential outcomes. These were then used in three rounds of a modified e-Delphi exercise with a panel of older people and housing professionals (*n* = 74) to test meanings and identify areas of agreement and disagreement. Descriptive statistics were used to present findings from previous rounds.

**Results:**

The survey confirmed a wide range of possible outcomes considered important. Through the e-Delphi rounds, panellists prioritised outcomes relating to living at home that could be influenced by design, and clarified their understanding of the meanings of outcomes. In subsequent rounds, they commented on earlier results. The exercise enabled five key outcome areas to be identified – staying independent, feeling safe, living in an adaptable home, enabling physical activity and enabling enjoyed activities- which were then tested for their content and applicability in panellists’ views.

**Conclusion:**

The five key outcome areas appeared meaningful to panellists, whilst also demonstrating nuanced meanings. They indicate useful outcomes for future research, though will require careful definition in each case to become measures. Importantly, they are informed by the views of those most immediately affected by better or poorer home design.

**Supplementary Information:**

The online version contains supplementary material available at 10.1186/s12877-024-05085-z.

## Background: The significance of home design for healthy cognitive ageing and its outcomes

There is emerging international agreement that good design of living environments can support people living with dementia to live better, and to enjoy improved quality of life, both for themselves and for those who support and care for them. Fleming et al. have recently explored the extent of agreement on the values and principles that it is believed should underlie such design [1]. They found general consensus that good design must respect the dignity, autonomy, independence, equality of opportunity and non-discrimination of people living with dementia.

In the light of this agreement and multiple experiments developing design supportive for cognitive ageing and dementia, a record of clear evidence demonstrating agreed outcomes might be expected. However, an extensive literature review [2] found key shortcomings in published research. These include a focus on small-scale, experimental design work, a predominance of research in communal settings rather than community-based homes, and failure to consult with and include the perspectives of key stakeholders including people ageing with cognitive change and professionals involved in building and/or supplying homes. More recently, Bowes et al., identified and reviewed 47 publications which had evaluated aspects of home design for living with cognitive change or dementia in the community [3].

This paper examines one of the key gaps that emerged from that review [3], namely the lack of understanding of and consensus about outcomes. Using a modified e-Delphi method, we explore the views on desirable outcomes of a range of professionals with housing-related roles and older people, and examine their meanings. Our aim is to clarify the range of understandings that exist, and arrive at a set of outcomes that make sense for both older people and professionals and that can be used in subsequent research. The exercise is one component of a large scale research project, Designing Homes for Healthy Cognitive Ageing (DesHCA) which aims to develop evidence-based, co-produced home designs which support people as they age with cognitive change, including dementia, and which are aesthetically appealing, practical, affordable and scalable.

Previous evaluations reviewed had used a wide range of outcomes [3], which had, in the large majority of instances, been defined by researchers. The five most widely used outcome measures were of the acceptability of the intervention, its usefulness, its impact on falls and/or risk of falls, activities of daily living and physical functioning (such as mobility). Studies which used multiple measures appeared more effective in terms of identifying pros and cons of design features.

There was a notable lack of use of outcomes defined by stakeholders. In particular, there were few studies that examined the views of older people living with cognitive change. Where this was attempted, several important findings emerged. They included the potential of a more qualitative approach to understand effects of design in the context of people’s lives [4]; the potential for perspectives to evolve over time [5]; and the diversity of older people’s perspectives [6]. Rarely considered outcomes that emerged in some cases included aspects of sociability, contacts with others, friendships and kin relationships [7–10]. However, the conclusions on outcomes that we were able to draw from the review remain suggestive, and the need for a more systematic understanding is clear.

A further area that had not been considered in the published evaluations was that of the wider system in which design innovations occur. Discussion of implications for the housing sector, including the commercial and public sectors was largely absent, emphasising the focus on small scale developments and experimental innovations. This area too, we suggest, requires further examination.

## Methods

The research aims to understand what outcomes professionals and older people feel are important as markers of successful home design for healthy cognitive ageing, including for people living with dementia. We also seek to develop improved understanding of how the outcomes are understood and prioritised by different stakeholders. Our aim is not to achieve a complete consensus, but to identify outcomes that can reasonably be seen as both significant and meaningful across stakeholder groups.

We adopt a modified e-Delphi approach. There is little agreement in the literature on the exact specification of Delphi approaches (e-Delphi being the exercise conducted electronically), despite general agreement that they seek to identify and understand consensus of opinion. Duncan et al. note the frequent use of the terms ‘modified Delphi’ or ‘using a Delphi approach’ [11] (p.2). Responding to this, Niederberger and Spranger, whilst arguing that there is a need for further methodological clarification, identify key content for rigorous reporting of Delphi exercises [12]: in the paper, we follow their guidance in presenting our study and use the term ‘modified e-Delphi’ to reflect the lack of methodological consensus.

Delphi methods combine qualitative and quantitative data elements across multiple iterations of asynchronous consultation with invited participating ‘experts’ to facilitate informed discussion and move towards identifying consensus views. Competing alternatives are proposed, discussed, and reformulated or discarded until opinions on the ‘best’ alternatives converge. They have been successfully employed in a variety of contexts, e.g. in the development of an assessment tool for medical procedures [13], selection of variables for public transport research [14], and design and validation of a questionnaire about digital competence [15].

Delphi methods, and particularly ‘e-Delphi’ consultations conducted electronically, have potential advantages over other consultative research methods. Anonymised, indirect interaction between participants allows panellists to contribute freely without concern for perceived rank or status; asynchronous consultation allows for a wider pool of potential participants and provides space for panellists to reflect before responding; and all panellists have equal opportunity to contribute to discussion, minimising ‘dominant voice’ bias which can occur in synchronous settings, such as focus groups. At a practical level, Delphi methods provide researchers with greater control of participant recruitment and the timetable for data collection whilst meaningfully involving participants whose personal circumstances or job roles may mean that their availability for scheduled interaction changes at short notice over the data collection period.

These characteristics of our approach were especially important given the range of participants, some of whom were in senior, powerful positions and others of whom seldom have the opportunity to inform research.

### E-Delphi: understanding outcomes

Our modified e-Delphi followed a multiple step process. An initial questionnaire provided data which then informed three rounds of interaction with our subsequently recruited e-Delphi panel.

The exercise began with an online, open-ended qualitative questionnaire^1^ (n = 62) that generated a wide range of potential outcomes. Respondents were recruited via social media and snowball sampling via networks with a view to ensuring maximum participant diversity and variety of views. UK-based older people (aged 55+) and professionals in the housing sector were targeted, with respondents being asked to identify themselves as ‘Expert by experience’, i.e. a person who owns, rents, or occupies a home (n = 31), or ‘Expert by profession’ (*n* = 31), i.e. someone who is engaged or involved in a housing-related field. The composition of the respondents to the questionnaire is shown in Table 1, which indicates a wide range of characteristics in both categories: our aim was to garner diverse responses from a varied population.

**Table 1:** Characteristics of questionnaire respondents

Older people (*N*)		Housing sector professionals (*N*)	
**Sex**		**Sex**	
Male	8	Male	11
Female	23	Female	20
**Age (years)**		**Age (years)**	
55–65	13	26–45	1
66–75	13	46–65	25
75–85	4	66–75	3
86+	1	75–85	2
**Health**		86+	0
Very Good	6	**Profession**	
Good	15	Design	5
Fair	7	Construction	1
Bad	3	Housing supply	8
Very Bad	1	Policy	5
**Long term illness**		Other	12
Yes	24	**Organisation type**	
No	8	Profit	9
Unknown	0	Public	12
**Activity limitation**		Non-profit	9
Severe	3	Other	1
Slight	17	**Role type**	
None	12	Strategy	13
**Memory difficulty**		Management	6
Unable to remember	1	Operations	6
A lot	2	Other	6
A little	7	**Experience length**	
No difficulty	22	0–5 years	1
**Tenancy**		6–10 years	4
Owned outright	20	11–15 years	4
Owned with mortgage	3	16 + years	22
Rented (council or HA)	8	**House type**	
**House type**		Detached	2
Detached	8	Semi-detached	5
Semi-detached	6	Terrace	2
Terrace	3	Flat	1
Flat	4	Unknown/not specified	21
Unknown/not specified	11	**Home occupancy**	
**Home occupancy**		Alone	5
Alone	9	With partner	3
With partner	6	With children	2
With children	5	With relatives	1
Unknown/not specified	12	Unknown/not specified	12

There are limitations in this sample of older people, despite its diversity in many respects. Conducting the survey online, whilst an economical method, inevitably excludes those who may be digitally excluded, who may include those in the oldest age groups [16]. The survey was also unable to capture socio-economic status or ethnic diversity with any reliability. Housing tenure however, a potential proxy for socio-economic status, was roughly equivalent to the general UK population in which just over one third (35.7%) are renters [17].

**Fig. 1:**
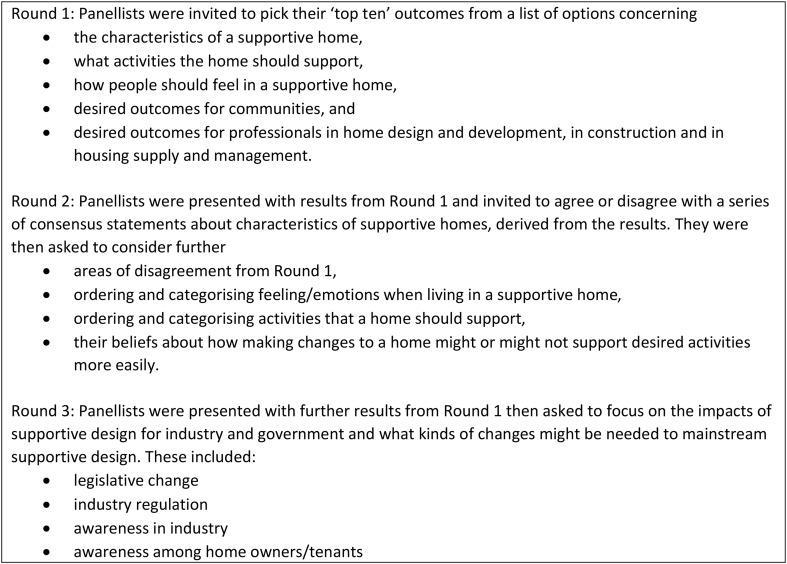
e-Delphi rounds

The questionnaire responses were then used, in the context of relevant literature, as the basis for tasks presented to the e-Delphi panel in three rounds. Figure 1 indicates the content of each round. It should be noted that Round 1 results were used as stimulus material in both Rounds 2 and 3.

Our e-Delphi panellists – experts – in the three rounds included individuals aged 55 + and professionals in design, construction, supply and management of housing. They were recruited via existing networks of contacts with organisations, both professional and community based, and by promoting the exercise via social media. Inevitably, an e-Delphi exercise excludes people who lack digital access. To guard against this potential source of bias, we provided an option for people without internet access to participate on paper or with equipment provided by us, but these options were not taken up and all contact with participants was via e-mail.

Table 2 indicates the composition of the panel, and their participation in each round of the e-Delphi exercise. The third category (professionals aged 55+) emerged from participant feedback – older professionals felt their combination of work and life experience gave them distinctive insights.

**Table 2:** e-Delphi participants Rounds 1–3

Category of participant	Individuals aged 55+	Professionals	Professionals aged 55+	Total
Round 1	22	28	24	74
Round 2	16	16	11	43
Round 3	11	7	9	27

We noted characteristics of the experts and this exercise demonstrated a reasonably diverse panel. The panels were mixed gender, though majority female. Professional experience was varied, including architects, designers, housing providers, lawyers, occupational therapists, physiotherapists, planners, service commissioners. Across the professionals and older people, several identified themselves as unpaid carers. As in the online survey, we were unable to capture ethnic or socio-economic diversity.

The table indicates drop off between rounds. This is usual for such exercises, especially where the questionnaires are long and the tasks may be seen as onerous. Shang notes that this is a classic issue for Delphi studies in which attrition may be as high as 92% [18]. We used many of the mitigating measures suggested by Shang including offering a non-electronic means of taking part, providing clarity on time commitment, purpose of study and a short (one month) time frame in which to complete the survey [18]. Whilst these did not prevent drop out, the evidence base suggests they will have mitigated it.

Descriptive statistics were used in presenting the results of each round to the participants, and these are also used in presenting the results of the exercise here. In presenting the results to participants, we used coloured bar charts and infographics at their request to make the results easier to understand. In Delphi studies, multiple different ways of understanding results and measuring consensus have been used [19]. As Diamond et al. [20] point out, setting agreement percentages in e-Delphi studies is an inevitably somewhat arbitrary process and the literature shows considerable variation. In the present study, we wanted to explore both agreements and disagreements among panellists, so we did not set a level of consensus that would be considered ‘acceptable’. Rather, in the presentation of results, we adopt a convention that 70% agreement is high, 50–69% medium and 30–49% low. We do not exclude factors where agreement is less than 30%, but rather examine them further, as we will describe. We set our ‘high agreement’ figure a little below the median 75% for agreement identified in Diamond et al.’s review [20], with the ‘medium’ and ‘low’ agreement levels set to incorporate most responses. Throughout, in calculating results, we have used mean values. Whilst some sources, such as Niederberger and Spranger recommend the use of medians [12], we found these did not provide sufficient differentiation between views, despite there being few outliers.

## Results

### Preliminary questionnaire: outcomes identified

The initial qualitative questionnaire included sections on: activities the home should facilitate; how the home should feel; benefits to communities; home design and development; the building industry; supply of homes; and management of homes. Respondents provided a long list of outcomes that they considered desirable in each of the categories. There were a number of responses that occurred repeatedly, and others that were less common, but often linked to some of the more frequently mentioned items. For example, in terms of activities, Table 3 illustrates the range of responses^2^.

**Table 3:** Range of responses to the question ‘What kinds of activities do you think homes could help with?’

Activity	Times mentioned
Garden(ing)	32
Personal care	28
Social activity	19
Accessibility	12
Community engagement/access	12
Exercise	12
Cooking	8
Digital connection	7
Physical movement	6
Independence	5
Care technology	4
Size of space	4
Access to bath, Activities, ADLs, IADLs, Crafts, Parking, Reading, Recreation, Warmth	3 each
Games, Mental health, Music, Normal life, Pets, Social eating	2 each
Clean, Community support, Learning, Plan for adaptation, Safety, Security, Smart home, Travel	1 each

Table 3 includes several examples of activities that might be considered similar, such as ‘exercise’ and ‘physical movement’ or ‘personal care’, ‘access to bath’ and ‘accessibility’. These data illustrate that different language may be used for similar activities, but also that attention needs to be given to the meanings of nominated items. Further examples can be viewed in the Supplementary Material which includes the full survey results.

### Round 1: Prioritising outcomes

The rather disparate lists were refined and used to ask the e-Delphi Round 1 panel to prioritise outcomes. Panellists were presented with a list of outcomes and asked to pick out their top ten (not ranked). The list used was informed by the pre-questionnaire, but adjusted to ensure that the responses were as clear and distinct as possible: for example, the ‘garden(ing)’ category was split into ‘spending time outside’ and ‘gardening’. Tables 4, 5 and 6 show the levels of agreement about supporting people as they age and Tables 7, 8 and 9 show the agreements about impacts for the community, the housing sector and health and social care. We present these results distinguishing the three self-identified expert groups: people aged 55+, professionals and professionals aged 55+.

**Table 4:** Top ten choices for what a home designed to support people as they age should be

Choices	Percentage including each choice in their top ten		
	Individuals aged 55+	Professionals aged 55+	Professionals
Designed for people with mobility impairments	82	96	82
Within walking distance of the community	95	63	75
Designed for people with cognitive change	68	75	82
Easy to keep warm or cool	82	71	75
Designed to make adaptation easier	77	54	89
Affordable	73	75	71
Easy to change to fit people’s preferences	68	75	71
A beautiful or modern home	59	71	46
In a community with people of different ages	59	46	68
Designed to reduce the risk and fear of falling	50	50	64
Fitted with easy to understand appliances	73	42	50
Designed for people with sensory impairments	45	46	61
A home anyone would want to live in	55	71	29
Within driving distance of the community	36	33	50
Designed to make installing telecare easier	41	42	39
Designed with ‘extra’ space	36	38	39
A haven or sanctuary	27	33	29
Equipped with Smart Home technologies	14	46	11
Built in a community for older people	9	13	7

In Table 4, a difference emerges in terms of prioritising conditions that can be supported: support for physical impairment scores higher than support for both cognitive and sensory impairment. Clear disagreements emerge relating to links to the community (‘in a community with people of different ages’ and ‘within driving distance of the community’) and technology use (‘designed to make installing telecare easier’ and ‘equipped with Smart Home technologies’). The table also suggests divergences of views in relation to aesthetics (‘a home anyone would want to live in’ and ‘a beautiful or modern home’), with older panellists (both professionals and non-professionals) seeming to value these more highly. Younger panellists were notably more focused on adaptability of the home. These aspects merit further investigation.

**Table 5:** Top ten choices for how people should feel when they live in a home designed to support people as they age

Choices	Percentage including each choice in their top ten		
	Individuals aged 55+	Professionals aged 55+	Professionals
Safe or secure	95	92	96
Independent	82	83	96
Able to change their home to suit their needs	86	71	79
Sociable, or connected with others	68	75	75
Happy or content	73	79	64
In control of their home	73	67	75
Part of their community	59	79	68
Supported or enabled	45	71	79
Warm	68	71	43
Financially secure	64	58	46
Like their home reflects their preferences or style	68	50	46
Like their home is a private space	59	46	46
Comfortable	50	54	43
Valued	27	42	46
Relaxed	45	13	32
Like their home is a space to have fun in	41	25	11
Fulfilled	9	13	21
Protected	9	13	18
Calm	23	0	14

Table 5 shows broad agreement across responses, though again, feeling ‘part of their community’ suggests some disagreement. Older people are more likely to nominate aesthetic aspects of the home (‘Like their home reflects their preferences or style’) as important with professionals (especially young ones) considering this less significant. There is apparently strong disagreement on feeling ‘supported or enabled’ with many fewer older people than professionals choosing that criterion. Older people however prioritise feeling ‘like their home is a space to have fun’ more highly than professionals.

**Table 6:** Top ten things that should be easier for people when they live in a home designed to support people as they age

Choices	Percentage including each choice in their top ten		
	Individuals aged 55+	Professionals aged 55+	Professionals
Staying independent	86	92	89
Staying physically active	82	79	82
Spending time outside	68	83	82
Keep doing the activities I enjoy	64	83	86
Bathing, showering and staying clean	68	71	86
Getting out and about in the community	55	79	75
Socialising with family and friends	59	75	57
Have a normal life as I get older	59	63	54
Preparing food and cooking meals	64	42	54
Staying safe	41	58	57
Having pets and animal companions	45	38	43
Going to the toilet	36	33	50
Housework and keeping the house clean	50	25	21
Using the computer/tablet or other technology	45	42	7
Using a car or mobility scooter	27	38	21
Gardening	41	8	29
Exercising	27	21	29
Having lunch or dinner with visitors	32	13	32
Getting dressed	23	8	36
Crafting hobbies (painting, knitting, model building etc.)	27	13	11
Making repairs and maintaining the house	23	17	11
Doing the laundry	27	4	14
Listening to music	18	8	7
Reading	14	4	7
Having a bath	9	4	7
Playing games and boardgames	9	0	4

Table 6 shows high levels of agreement for the top five items. Community links again engender disagreement, with older people seeing them as markedly less important than other items in the list of possible responses. Older people are more likely to value being able to maintain the home, prepare meals, do laundry and keep the house clean, emphasising aspects of ordinary life that professionals are less likely to select. They are also more likely to include crafting, hobbies and various sedentary leisure activities in their top ten than either professional category. Older people, including older professionals, include design making it easier to use a computer as desirable, whilst younger professionals do not see this as important. In this table, professionals seem to value the continuation of enjoyable activities, in contrast to their views about having fun indicated in Table 5. At the lower end of this table, there are some mixed results: this issue is picked up in Round 2.

**Table 7:** Top ten choices for ways building supportive homes might benefit the community

Choices	Percentage including each choice in their top ten		
	Individuals aged 55+	Professionals aged 55+	Professionals
Communities become more physically accessible	73	75	79
Different generations can learn from each other	68	50	86
Residents stay in their community longer	77	58	64
More intergenerational activities and spaces	50	63	79
Communities become more supportive	50	63	75
Community members become more connected	45	63	71
Local services are used more often	68	46	61
Communities become more inclusive	32	54	68
More activities offered in the community	36	50	57
Reduced pressure on public services	45	50	46
More demand for outdoor or green spaces	50	46	43
Community spaces are better maintained	45	29	50
Improvements to transportation	41	50	36
Community keeps a connection to its history	32	33	25
Communities become focused on older people	23	38	29
More community engagement	18	29	32
More opportunities for employment	18	33	29
Opportunities for older people to become mentors	18	17	43
More demand for shops and businesses	36	13	18
More demand for community spaces	36	17	11

Of the potential benefits of supportively designed housing to the wider community listed in Table 7, only physical accessibility of the community reaches a high level of agreement, followed by six factors reaching moderate agreement, though these still differ between groups. All the remaining factors have low levels of agreement. The issues emerging here are followed up in Round 2.

Table 8 summarises results concerning the perceived benefits of supported housing for housing design, construction, supply and management.

**Table 8:** Top ten ways that building supportive homes might benefit housing design, construction, supply and management

		N	Overall agreement	Individuals aged 55 + agreement	Professionals aged 55 + agreement	Professionals agreement
			Percentage (%)			
Design	Professionals learn more about the principles of supportive design, or designing for older people	70	96	86	100	96
	Designs for all houses improve	66	90	95	88	86
	Professionals learn more about what people living with conditions that lead to cognitive change (such as dementia, Parkinson’s or a stroke) need or want	66	90	77	92	96
	Older people can live for longer in the homes and communities of their choosing	65	89	77	96	89
	UK housing stock is improved	63	86	82	92	82
	Professionals learn more about what older people need and want	62	85	86	83	82
	Supportive houses reduce pressure on public services	61	84	73	83	89
	Supportive houses reduce care home admission	61	84	77	83	86
	More supportive housing creates a better future	58	79	59	79	93
	Supportive houses improve communities	52	71	64	54	89
Construction	Professionals learn more about the principles of supportive design, or designing for older people	69	95	86	96	96
	UK housing stock is improved	63	86	86	79	89
	Professionals learn more about what people living with conditions that lead to cognitive change (such as dementia, Parkinson’s or a stroke) need or want	61	84	86	88	75
	Older people can live for longer in the homes and communities of their choosing	60	82	68	83	89
	Guidance and regulations will improve	60	82	68	79	93
	Supportive homes will be more sustainable	58	79	68	79	86
	Professionals learn more about what older people need and want	56	77	77	71	79
	More supportive housing creates a better future	53	73	59	63	89
	Supportive houses reduce pressure on public services	51	70	55	75	75
	Supportive houses reduce care home admission	48	66	64	67	64
Supply	UK housing stock is improved	66	90	95	83	89
	Older people can live for longer in the homes and communities of their choosing	64	88	86	96	79
	Supportive homes will be more sustainable	62	85	86	79	86
	Supportive houses reduce pressure on public services	61	84	82	83	82
	Professionals improve their practice overall	61	84	82	75	89
	More supportive housing creates a better future	59	81	59	92	86
	Professionals learn more about what people living with conditions that lead to cognitive change (such as dementia, Parkinson’s or a stroke) need or want	59	81	68	75	93
	Professionals learn more about what older people need and want	58	79	77	75	82
	Communities become better and more supportive	58	79	73	79	82
	Communities become more inclusive	55	75	77	67	79
Management	Housing stock requires less adaptation in the future	72	99	100	96	82
	Housing stock becomes more flexible and suits more people’s needs	70	96	91	100	86
	Supportive houses reduce pressure on public services	67	92	86	92	79
	Older people can live for longer in the homes and communities of their choosing	67	92	82	100	86
	Supportive houses make it easier to provide residents with support	65	89	77	88	75
	More supportive housing creates a better future	62	85	64	88	75
	Professionals learn more about what older people need and want	59	81	91	75	64
	UK housing stock is improved	56	77	82	71	61
	Supportive houses are easier to manage	50	68	68	71	61
	Professionals learn more about what people living with conditions that lead to cognitive change (such as dementia, Parkinson’s or a stroke) need or want	50	68	59	71	61

Several factors appeared in the top ten across all four categories: design, construction, supply, and management. For example, more supportive housing creating a better future, older people being able to live longer in the place of their choosing, professionals learning more about what older people and people living with conditions relating to cognitive change need and want, reducing pressure on public services, and improvement of housing stock.

The results in Table 8 indicate high levels of agreement; across the listed factors overall agreement was 66% or higher. Slightly lower levels of agreement were observed within the individuals aged 55 + category, particularly for construction related factors.

For impact on the housing design and construction sectors, agreements centre around educating the sector about design and taking account of the views of older people. For impact on the supply of housing, panellists typically agreed that housing stock would be improved, homes would be more sustainable, and people could live in their homes longer. Finally, the impact on management suggested that homes would require less adaptations in the future, stock would be more flexible and reduce pressure on public services.

In the initial questionnaire, only eight potential benefits for health and social care of supportive housing had been identified. In itself, this is significant, as one of the key challenges in promoting the potential of supportive housing is that the transfer of benefits between sectors is not generally considered, and the budgets related to each sector are separate. Panellists were asked to rank the eight identified benefits, and we present results merged across categories due to the small number of options. Table 9 indicates the ranking of these eight.

**Table 9:** Ranking of ways that building supportive homes might benefit health and social care services

Rank	Choices
1	Supporting independence and improving people’s mental health and well-being
2	Reduce the number of people living in housing that does not suit them
3	Easier to provide care to someone living at home
4	Reduce the risk of hospital admission
5	Easier for people who are living with dementia or cognitive change to live in the home of their choosing for longer
6	Reduce the risk of people moving to a care home if they do not wish to
7	Easier for health and social care professionals to adapt homes for people’s needs later.
8	Reduce the risk of delayed release from hospital

In this ranking, the emphasis on independence and living a good life reflects earlier results, particularly regarding older people’s priorities.

Overall, the Round 1 results indicate an emerging consensus in five key areas: staying independent, feeling safe, living in an adaptable home, enabling physical activity and enabling enjoyed activities. Some areas of disagreement also emerge, including; issues relating to community outcomes, to technology, and to support for different types of impairments. We also note that professionals were less inclined than older people to prioritise aesthetic aspects of the home and improving the ease of aspects of ordinary life (such as hobbies, household tasks, sociability). The results relating to the sector and the impacts for health and social care show high levels of agreement on benefits.

### Round 2: Agreements and disagreements

In Round 2, panellists were presented with the results from Round 1, and asked to comment on whether their own views agreed with those of the whole panel, and what messages they took from the data. They were asked to identify what they thought could be achieved by building or adapting homes to make them more supportive for people getting older. This was done through agreeing or disagreeing with a series of statements about what conditions housing could support, about the use of technology and community-related items, all of which had engendered some disagreement in Round 1.

They were also asked questions designed to improve our understanding of the meanings which they ascribed to the most widely nominated desirable outcomes of supportive housing. In their qualitative comments on the results of Round 1, panellists expressed general agreement with the rankings. However, they also highlighted that some more specific items could be part of the larger categories – so, for example, ‘exercising’ could perhaps be incorporated into ‘physical activity’ among the activities that should be easier. These comments support the effort made in this round to identify the really core preferred outcomes and their meanings for respondents, providing an internal check of the process. To explore panellists’ understandings of relationships between different outcomes, the five widely supported outcome areas were used as ‘boxes’ into which panellists were asked to insert less popular items, if they felt these could be seen as ‘part’ of these larger categories. This process was intended to help clarify what the different groups involved understood by the various outcomes and to support the development of a widely understood and desired list of outcomes.

Table 10 shows the levels of agreement and disagreement across the panel groups regarding statements about what supportive design might be able to achieve in the contentious areas of support for different impairments, issues relating to technology and community aspects. We did not include an option for slight disagreement, as we were seeking to identify broad consensus, whilst also allowing people the option of more significant disagreement.

**Table 10:** Round 2 ideas about the effectiveness of supportive design issues

	Strongly Agree (*N*)					Somewhat Agree (*N*)					Neither Agree nor Disagree (*N*)					Strongly Disagree (*N*)				
	55+	Prof 55+	Prof	Total	% row	55+	Prof 55+	Prof	Total	% row	55+	Prof 55+	Prof	Total	% row	55+	Prof 55+	Prof	Total	% row
It is possible to design or adapt a home to support someone who has difficulty with their hearing or sight*	9	7	7	23	53*	7	9	2	18	42	0	0	0	0	0	0	0	2	2	5
It is possible to design or adapt a home to support someone who has difficulty with their mobility or movement**	10	12	9	31	72**	6	4	0	10	23	0	0	0	0	0	0	0	2	2	5
It is possible to design or adapt a home to support someone who has difficulty with their memory, thinking, or cognition	3	6	6	15	36	10	7	3	20	48	2	3	0	5	12	0	0	2	2	5
Homes that are designed to support people as they get older should have Smart Home technology installed from the beginning	3	9	2	14	33	4	2	6	12	28	7	4	3	14	33	2	1	0	3	7
Homes that are designed to support people as they get older should make it easy to install remote care systems (like telehealth, or telecare) at a later date	4	11	6	21	49	10	2	3	15	35	2	2	1	5	12	0	1	1	2	5
Homes that are designed to support people as they get older should always be fitted with appliances that are easy to understand**	13	12	7	32	74**	3	0	1	4	9	0	3	1	4	9	0	1	2	3	7
Homes that are designed to support people as they get older should be built in places that make it easy to access the community by car	1	2	1	4	9	7	5	3	15	35	7	7	5	19	44	1	2	2	5	12
Homes that are designed to support people as they get older should be built in places that make it easy to access the community by public transport*	12	11	6	29	69*	3	2	2	7	17	1	2	1	4	10	0	0	2	2	5
Building homes that support people as they get older will lead to more demand for community spaces	4	1	1	6	14	6	5	7	18	43	6	8	2	16	38	0	1	1	2	5
Building homes that support people as they get older will lead to more demand for local shops and businesses	7	4	3	14	33	8	7	5	20	47	1	5	2	8	19	0	0	1	1	2
Building homes that support people as they get older will lead to people being more involved in their community	6	5	4	15	35	6	7	4	17	40	4	4	3	11	26	0	0	0	0	0

*Notes* 55+: individuals aged 55+, Prof 55+: professionals aged 55+, Prof: professionals

Overall, Table 10 shows little strong disagreement with any of the statements, though the degree of consensus on possible outcomes remains variable. The areas of strongest agreement (marked ** – ≥70% strong agreement) relate to the potential for design to support physical mobility and the importance of incorporating easy-to-understand appliances. Weaker consensus (marked * − 50–69% medium agreement) is also shown regarding the importance of access to the community by public transport and the potential for design to support sensory impairment. Statements with less support include the potential for design to support cognitive impairment: whilst our panellists clearly see this as desirable, they are less convinced of its potential successful implementation, with 17% in total either disagreeing or expressing a neutral view of this, including two professionals who expressed strong disagreement. This finding adds weight to the suggestion in Round 1 that, even where there is awareness of the existence of design to support cognitive ageing, its potential is not necessarily well understood.

Table 10 also includes several findings that raise potential issues for further investigation. These include neutrality or disagreement regarding the use of smart technology (40%); car use to access the community (56%); potentially increased demand for community spaces (43%); potentially increased demand for local shops and businesses; potentially increased involvement of older people in their communities (26%). Also, within one of the areas of strong agreement, regarding appliances that are easy to understand, 17% are either neutral or disagree. These cannot be explained from the data, especially in the light of the small samples.

**Fig. 2:**
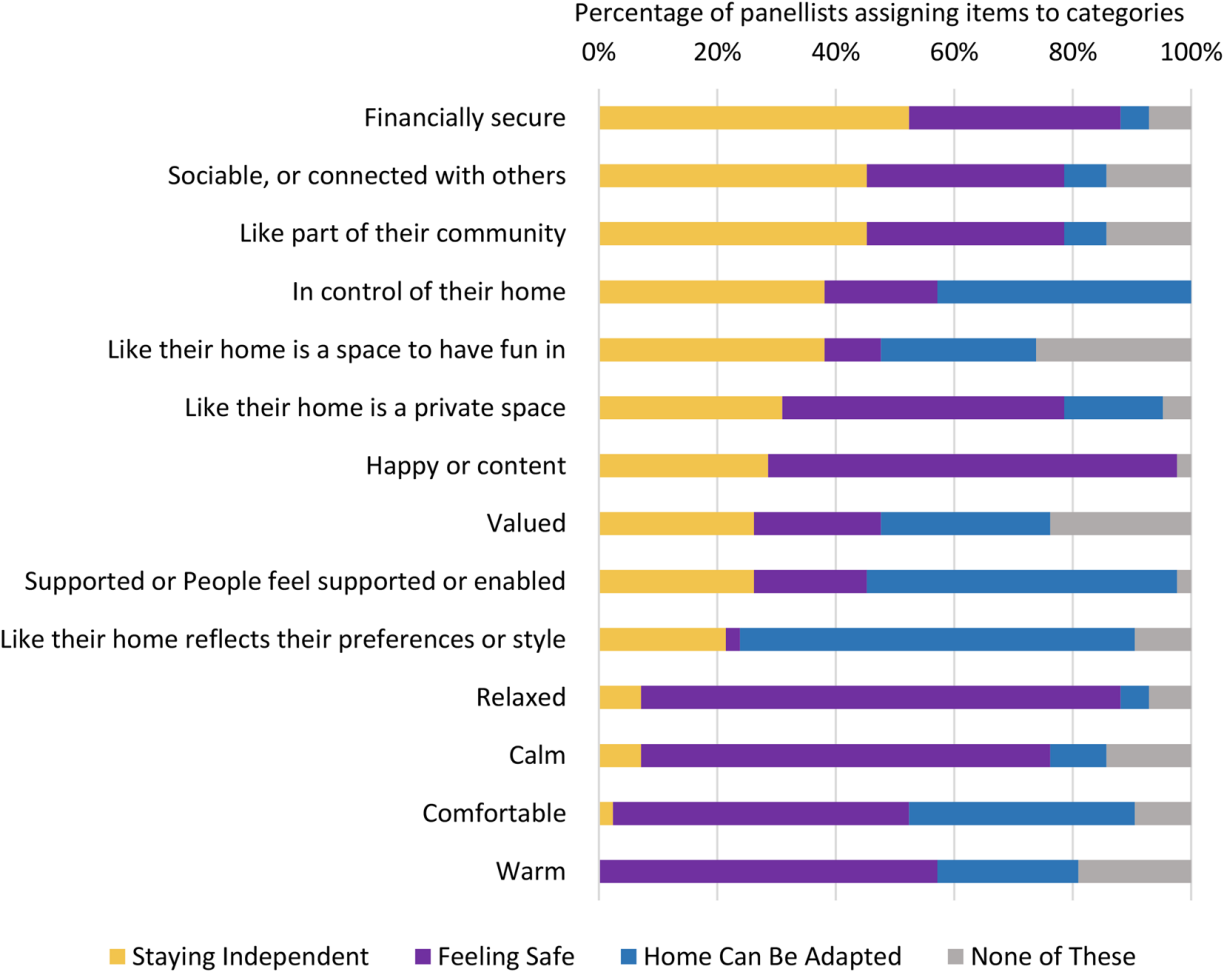
Clarifying feelings considered desirable

**Fig. 3:**
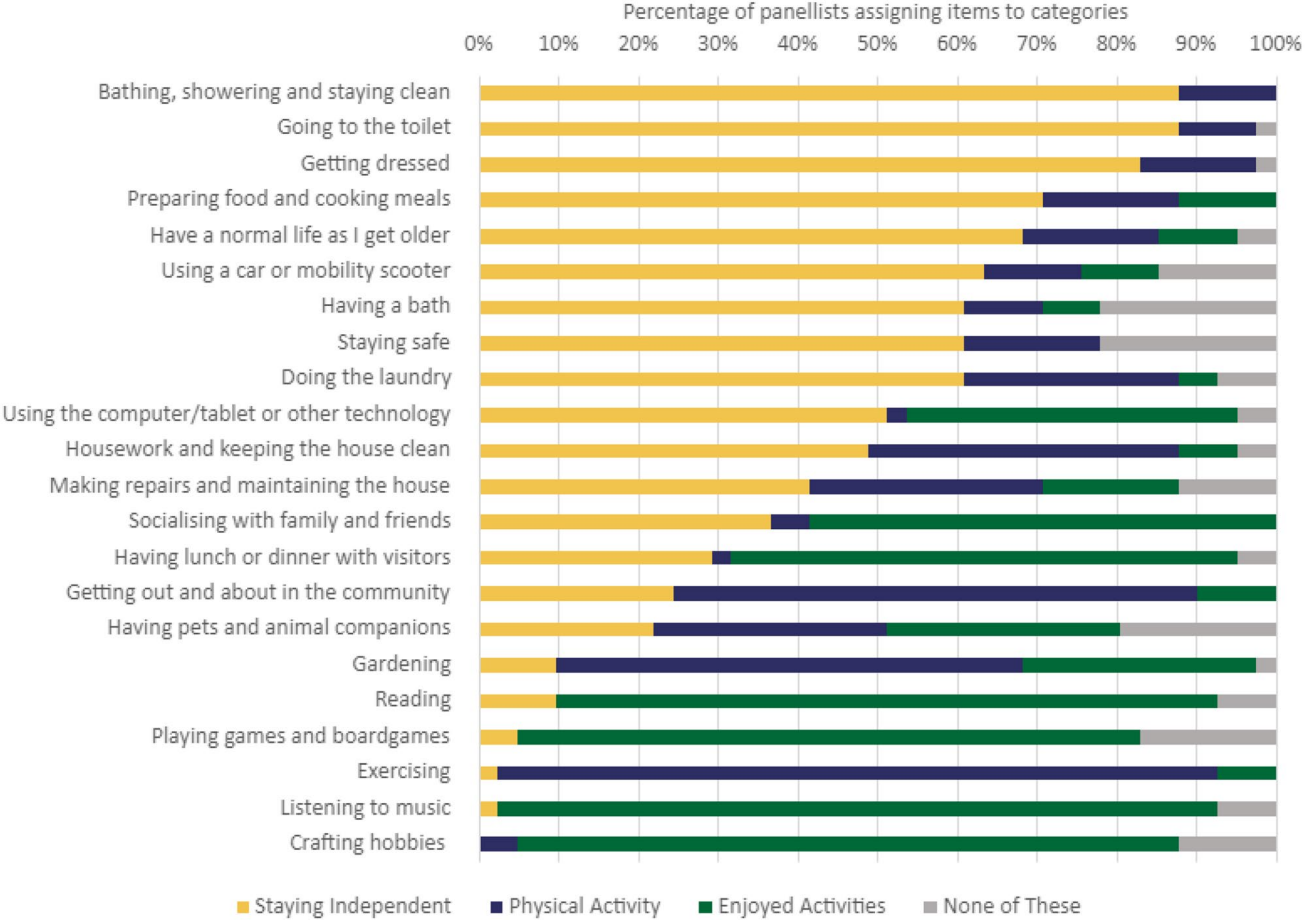
Clarifying activities considered desirable

The panellists were asked to clarify how the less popularly identified activities might be incorporated into the five key areas of agreement, derived from the most popular responses in Round 1. Their responses, shown in Figs. 2 and 3, indicate the emergence of a consensus across the panels about broad categories of outcome considered important in relation to feelings and activities, and how the individual activities explored related to three broad consensus categories. Importantly, the final column (‘none of these’) shows continuing debate in some areas and emphasises the need to understand the nuances of meaning across the outcome categories. Furthermore, these findings show the complexity of the categories which indicate broad agreement. They have multiple components, and some feelings and activities may be seen as linked with more than one category. For example, ‘Being in control of one’s home’ crosses three categories.

Overall, findings from Round 2 confirm the complexities of meanings in the broad categories of outcomes, but also demonstrate consensus that panellists found them meaningful.

### Round 3: Deepening understanding

Round 3 also explored further the disagreements that had emerged in Round 1 concerning different categories of panellists’ ideas about the wider impacts of supportive design. Panellists were again presented with Round 1 results. The round examined panellists’ perspectives on the potential impact of supportive home design and adaptation on the housing sector across the UK, with a focus on specific changes that they felt might make a difference positively or negatively, including changes to government legislation, industry regulations, increased awareness within industry, and raised public awareness. Table 11 indicates the responses to potential areas of change that participants thought might make a difference.

**Table 11:** How would change in particular areas help to encourage more supportive housing? (*N* = 27)

Potential change	% of all panellists responding that changes would be positive in different sectors of housing			
	Design	Construction	Supply	Management
Government legislation	100	86	90	87
Industry regulation	95	65	80	86
Raising industry awareness	100	95	100	100
Raising public awareness	100	91	86	95

These responses showed high levels of agreement across everyone in the panel, but in the light of the more nuanced views below, it was clear that whilst desirable, changes in these areas were felt to be challenging to achieve. The results suggest less confidence (65%) regarding the potential impact of regulation for the construction sector.

In Round 1, professionals under the age of 55 had been less likely to choose options about new job opportunities, new contracts, or new opportunities for profit among their ‘top ten’ benefits for building more supportive homes than those in other groups. They were also less likely to choose positive PR or reputation as a major benefit of building more supportive homes. This pattern was repeated across different areas of the industry.

To explore some reasons why these differences might exist, we asked Round 3 panellists to identify whether parts of the sector would benefit from building more supportive homes in terms of reputation, attracting more business, gaining access to customers or markets, job opportunities for professionals and better job satisfaction for professionals. Table 12 shows the results of these questions (percentages are not provided due to the low number of panellists at this stage).

**Table 12:** Potential impact of change for different parts of the sector

	**Design professionals**				**Building and construction professionals**			
	**55+**	**Prof 55+**	**Prof**	**Total**	**55+**	**Prof 55+**	**Prof**	**Total**
Better reputation for organisations	9	6	5	20	8	4	5	17
More business	6	4	3	13	6	2	4	12
Access to markets	5	8	3	16	6	8	3	17
More job opportunities	5	5	3	13	6	6	4	16
Job satisfaction	8	9	4	21	6	8	3	17
	**Supply of housing**				**Management of housing**			
	**55+**	**Prof 55+**	**Prof**	**Total**	**55+**	**Prof 55+**	**Prof**	**Total**
Better reputation for organisations	8	8	5	21	7	6	5	18
More business	6	3	3	12	5	6	3	14
Access to markets	6	7	3	16	5	6	3	14
More job opportunities	7	6	3	16	5	7	4	16
Job satisfaction	7	7	3	17	6	9	4	19

*Notes* 55+: Individuals aged 55+, Prof 55+: professionals aged 55+, Prof: professionals

Overall, and with the caveat that numbers of panellists had reduced for this round to 27, Table 12 suggests higher agreement about impacts in terms of reputation, access to markets and job satisfaction across the sector, and lower agreement about whether business or job opportunities might be affected by implementing supportive design.

Panellists were invited to add further qualitative comments to their initial responses, and these revealed nuances reflecting perceptions of the challenges involved in making changes.

In terms of improving the reputation of organisations, several panellists commented that ‘housing is all driven by profit’, seeing this as an obstacle to ensuring supportive provision. It was also highlighted that housing providers wish to appeal to different markets, and that for example younger families might not find features of supportive housing attractive. One participant noted that much so-called ‘supportive housing’ was built to a formula, and provided only limited support, with pressure on residents to move on if their support needs increased. This emphasised the importance of providing good quality supportive housing, but the difficulties of doing this were linked to incentive systems which were considered not to align with best practice. Older professionals had less positive views about organisational reputation, with other professionals and older people being more optimistic.

The older professionals were also pessimistic about increasing business and access to markets. They felt that financial issues would drive access to business, with investment being made in areas seen as most profitable. In the context of competing interests, supportive housing might be seen as more expensive and a less attractive business proposition than for example student accommodation. There was caution about building homes targeting specific markets, though it was pointed out that many supportive housing features, such as level thresholds, were both unobtrusive and universally useful. There was also scepticism about some social housing providers, who had positioned themselves as offering supportive housing, but who were perceived to opt consistently for the cheapest and most basic materials and design features.

Considering job opportunities and job satisfaction, comments focused on the likelihood not of new opportunities but of changing opportunities with re-focused roles, including training professionals in supportive design. A shortage of relevant expertise in the social housing sector was noted.

### Discussion: Consensus on outcomes?

The modified e-Delphi study aimed to identify desired outcomes of cognitively supportive home design that could be used by researchers to develop measures that were meaningful for older people and professionals in housing. The exercise has developed deeper understanding of preferred outcomes for supportive housing design. There is key learning for research which seeks to use effective and meaningful outcome measures regarding the implementation of supportive home design.

In reference to the outcomes considered in previous literature discussed earlier in the paper [3], the exercise demonstrates that activities of daily living and physical functioning are considered important, but falls do not figure as a separate category of outcome. The conclusion drawn from previous literature [3], that the use of multiple outcome measures seems to produce more meaningful results, is supported by the evidence from the e-Delphi exercise that the categories of outcome often have many components and may be understood in different ways. Whereas the literature suggested that stakeholder-defined outcomes were important, and our data confirms that these can be valuable, we also find that they are complex, and that any study would need to define them carefully. We are not arguing that outcomes used in earlier literature should be abandoned: the implication of our work is that multiple outcomes are likely to be important, and that their clarity and purpose need to be improved.

Regarding older people’s and sector professionals’ perspectives, we found few consistent differences, especially between older people and the older professionals. However, younger professionals on occasion appeared to be using stereotypes of older people – notably that they do not use technology or have hobbies or want to enjoy life. Particularly striking was the preference of older people to live in a home that would appeal to anyone, and the professional tendency not to consider aesthetics as a priority. The thinking about the impacts of improved design for the sector was less clear: this may help account for its lack of consideration in previous literature [3], in that the questions are complex and the impacts difficult to define. Identifying outcomes that help evaluate the impacts for the sector is however important if scalability issues are to be addressed.

As the exercise progressed, a number of key conclusions and implications emerged. Firstly, the variety of outcomes identified in the pre-questionnaire is important and alerts us to the need for clarity in expressing these. This theme recurred throughout the exercise as we explored the range of interpretations of different outcomes. The range of outcomes considered important is much wider than used in previous literature [3] and includes an emphasis on the benefits desired by older people themselves.

Secondly in Round 1, it became clear that different groups of experts may prioritise or highlight different design features as desirable outcomes with, for example, older people more likely to prioritise ‘easy to understand appliances’ and professionals seeing smart technology as important. We do not conclude that the most popular items should be selected for inclusion in designs, or that less popular items should be automatically excluded, due to the different understandings of items identified, and to the evidence that preferences can differ significantly. In decision making processes about home design, collaborative work involving both older people and professionals is likely to engender decisions which produce genuinely supportive homes in a context of flexible design.

Thirdly, we were able to identify strong agreement in some areas of priority in Round 1, including safety and security, independence and physical activity. Also, some areas were seen as less likely to be impacted by supportive housing, notably aspects relating to community access and involvement, though there was broad consensus that this was a desirable outcome.

Fourthly, the deeper exploration in Round 2 enhanced understanding of what were the underlying key factors, rather than the perhaps superficial particularities of desired and desirable outcomes, demonstrating how some of the very detailed items mentioned could be seen as contributing to underlying and more fundamental outcomes. This is reminiscent of Pawson’s discussion of ‘latent mechanisms’ [21], processes that underlie the surface satisfaction factors and start to reveal what works, for whom and how. The Round 2 analysis grouped the outcomes into broad categories, to which the more detailed items contributed. These overarching outcomes were staying independent, physical activity, enjoyment, feeling safe, and having an adaptable/flexible home that could be changed to provide support as needed. Each of these had multiple aspects, and some aspects contributed to more than one of the larger outcomes. For example, bathing, showering and staying clean were mostly linked to staying independent, but also contributed to physical activity. Having pets and animals was seen as contributing to staying independent, physical activity and enjoyment. Importantly therefore, we are able to identify complex, multifactorial outcomes, and demonstrate how they may consist of several different aspects and activities.

Fifthly, in terms of thinking about scalability, the widespread agreement about the need for systemic change is significant, highlighting challenges for delivery of supportive housing at scale. The nuanced views about the impacts for the sector of delivering supportive housing emphasise the complexity of these challenges, highlighting competing imperatives and interests.

The work has some key limitations, including some relating to the method. First, the sample of respondents, though diverse and informative, remained small, and conclusions are therefore suggestive rather than definitive. As noted, we were unable to consider socio-economic status and ethnic diversity, and both are areas which would merit further investigation. Of the initial 31 older people in the e-Delphi panel, nine reported memory difficulty of any kind. The lifetime risk of becoming a carer of someone with dementia or of developing dementia (of which memory issues are only one symptom) has recently been calculated as 55% [22], further work with people experiencing cognitive change would enhance our findings. Second, and as is common with Delphi exercises, there was a drop out between rounds, despite recommended measures being taken to minimise this. Thirdly, the exercise has raised new questions about what outcomes need to be considered in evaluating supportive design for healthy cognitive ageing: alone therefore, this part of the study provides indicative findings. These will be complemented as other elements of the DesHCA research are published.

## Conclusion

Earlier research produced a limited picture of outcomes of cognitively supportive housing that had been or could be used in evaluating home designs. Despite suggestions that complex, stakeholder-defined outcomes were needed, it was not possible to identify these from the literature. In particular, researchers had provided limited discussion of cognitively supportive housing in the context of the housing sector. Our e-Delphi exercise aimed to address these gaps by exploring how a panel of varied stakeholders identified and understood potential outcomes of cognitively supportive home design. The research enabled us to identify five key outcomes in the form of ‘latent mechanisms’ that can provide reference points for future evaluations: staying independent, physical activity, enjoyment, feeling safe, and having an adaptable/flexible home that could be changed to provide support as needed [21].

The significance of improving understanding of outcomes is supported by the panellists’ apparently limited confidence in the potential for home design to support healthier cognitive ageing, which suggested that they too were perhaps unsure about possible outcomes. When prompted by our questions however, they enabled us to understand what was likely to be important for different stakeholders and to identify the framework of key considerations within which particular items could be better understood. The findings emphasise the importance for future research of clarity about outcomes, particularly where different understandings could come into play.

Scalability is a continuing challenge: in this area, our work has brought to the surface potentially competing imperatives and interests, which may prove obstacles to implementation. Further work is required in this area: elsewhere we have developed a theory of change which sets out the challenges and the pathways to implementation and achievement of outcomes, informed by the e-Delphi exercise [23].

### Electronic supplementary material

Below is the link to the electronic supplementary material.

Supplementary Material 1

Supplementary Material 2

Supplementary Material 3

Supplementary Material 4

Supplementary Material 5

Supplementary Material 6

## Data Availability

The datasets generated and analysed during the current study are available from the corresponding author on reasonable request. Following completion of the DesHCA project, the data will be permanently archived. Please contact the corresponding author for details.

## Footnotes

1 All questionnaires are available in the supplementary material.

2 Full results are in the Supplementary Material.
