# Supplementary material for: Outcomes of home design to support healthy cognitive ageing: modified e-Delphi exercise with older people and housing-related professionals
Source: BMC Geriatr. 2024 Jun 24;24:546. doi: 10.1186/s12877-024-05085-z (PMC11194886; doi:10.1186/s12877-024-05085-z)
Supplement: Supplementary file 1 — Supplementary Material 1 [file 12877_2024_5085_MOESM1_ESM.docx]

# Supplementary material: Results of pre survey

This tables provide the full range of responses to the pre-survey. Table S1 is also included in the main text as an example of these results.

Table S1 (same as Table 3): Range of responses to the question ‘What kinds of activities do you think homes could help with?

| **Activity** | **Times mentioned** |
| --- | --- |
| Garden(ing) | 32 |
| Personal care | 28 |
| Social activity | 19 |
| Accessibility | 12 |
| Community engagement/access | 12 |
| Exercise | 12 |
| Cooking | 8 |
| Digital connection | 7 |
| Physical movement | 6 |
| Independence | 5 |
| Care technology | 4 |
| Size of space | 4 |
| Access to bath, Activities, ADLs, IADLs, Crafts, Parking, Reading, Recreation, Warmth | 3 each |
| Games, Mantal health, Music, Normal life, Pets, Social eating | 2 each |
| Clean, Community support, Learning, Plan for adaptation, Safety, Security, Smart home, Travel | 1 each |

Table S2: Range of responses to the question ‘How do you think ‘home’ should make a person feel?’

| **Feeling** | **Times mentioned** |
| --- | --- |
| Safe | 43 |
| Comfortable | 21 |
| Content/Happy/Positive | 17 |
| Enabling | 13 |
| Warm/Temperature controlled | 12 |
| Connected | 10 |
| Personalised | 8 |
| Independent | 6 |
| Affordable | 6 |
| Maintained | 4 |
| Beautiful | 4 |
| Accessible | 4 |
| Secure, In control, Relaxed, Flexible, Supported, Social, Haven/Sanctuary | 3 each |
| Community engagement/access, Quiet, Private | 2 each |
| Engaged, Welcoming, Fulfilled, Garden/open space, Modern, Size of space, Usable, Calm, Fun, Freedom, Valued, Consistent, Protected, Spacious | 1 each |

Table S3: Range of responses to the question ‘In an ideal world, how would this housing benefit local neighbourhoods or communities?’

| **Benefit to communities** | **Times mentioned** |
| --- | --- |
| Community engagement | 35 |
| Social connection | 13 |
| Intergenerational activities/spaces | 12 |
| Retaining residents | 11 |
| Inclusive community | 10 |
| Accessibility | 9 |
| Services | 8 |
| Shops/businesses | 7 |
| Community spaces | 6 |
| Green spaces | 5 |
| Support | 4 |
| Activities | 3 |
| Connections to history, Education, Exclusive community, Mentoring | 2 each |
| Connection to community, improved community, Transportation, Maintained | 1 each |

Table S4: Range of responses to the question ‘What benefits might there be for those involved in the design and development (e.g. architects, designers, developers) of housing which supports healthy cognitive ageing?’

| **Benefit to housing design/development** | **Times mentioned** |
| --- | --- |
| Improve housing stock | 13 |
| Improve understanding | 12 |
| Understand needs | 9 |
| Improve design | 7 |
| Job satisfaction | 7 |
| Market/Profit | 6 |
| New build opportunities | 4 |
| Better future | 4 |
| Retain residents | 4 |
| Reduce pressure on public services | 3 |
| Improve communities, Reduce care home admissions, Positive PR | 1 each |

Table S5: Range of responses to the question ‘What benefits might there be for individuals, organisations or bodies with roles in housing construction (e.g. building and construction professionals, building control) involved in the development of housing which supports healthy cognitive ageing?’

| **Benefit to housing construction** | **Times mentioned** |
| --- | --- |
| Improved practice | 7 |
| Job satisfaction | 7 |
| New builds/Business | 7 |
| Improved understanding | 6 |
| Market/Profit | 6 |
| Improve housing stock | 3 |
| Employment opportunities, Reduce pressure on public services, Sustainability | 2 each |
| Better future, Improved guidance, Increased business, Positive PR, reduced care home admission, Retain residents | 1 each |

Table S6: Range of responses to the question ‘What benefits might there be for individuals, organisations or bodies involved in supplying housing (e.g. house-builders, housing associations, local authorities) which supports healthy cognitive ageing?’

| **Benefit to housing supply** | **Times mentioned** |
| --- | --- |
| Improve housing stock | 12 |
| Market/Profit | 11 |
| Reduce pressure on public services | 8 |
| Retain residents | 8 |
| Improve understanding | 6 |
| New build opportunities | 4 |
| Better Future, Improved practice, Job satisfaction | 3 each |
| Employment opportunities, Inclusive community, Positive PR | 2 each |
| Improve communities, Sustainability | 1 each |

Table S7: Range of responses to the question ‘What possible benefits or positive changes might there be for individuals, organisations or bodies that manage housing (e.g. housing associations, local authorities, landlords) which supports healthy cognitive ageing?’

| **Benefit to housing management** | **Times mentioned** |
| --- | --- |
| Easier to manage | 13 |
| Reduce pressure on public services | 13 |
| Market/Profit | 10 |
| Improved understanding | 8 |
| Retain residents | 6 |
| Improved housing stock | 5 |
| Job satisfaction | 4 |
| Improved practice | 3 |
| Better future, Employment opportunities, Flexible stock, Future proofing | 1 each |
