# Supplementary material for: Outcomes of home design to support healthy cognitive ageing: modified e-Delphi exercise with older people and housing-related professionals
Source: BMC Geriatr. 2024 Jun 24;24:546. doi: 10.1186/s12877-024-05085-z (PMC11194886; doi:10.1186/s12877-024-05085-z)
Supplement: Supplementary file 2 — Supplementary Material 2 [file 12877_2024_5085_MOESM2_ESM.pdf]

# DesHCA consultation Questionnaire October 2022

## About this questionnaire and your responses to it

**This questionnaire is part of the DesHCA research project at the University of Stirling.**

DesHCA (Designing Homes for Healthy Cognitive Ageing) aims to design homes and make changes to home design which will support people to live better as they age. The designs we produce will be directly informed by the views of older people themselves and those involved in designing, building, and providing homes.

We are circulating this questionnaire widely to gather views about what the benefits of housing that supports healthy cognitive ageing could be. When we have collected a range of views, we will bring these together and examine where there is agreement or disagreement in opinions. At the end of this questionnaire, we will ask you whether you would like to help us with that part of our work.

This questionnaire should take no more than 30 minutes to complete.

**Your participation is completely voluntary. You do not have to complete this survey or answer any questions within it if you do not wish to do so. Your contribution is completely anonymous unless you choose (at the end of the questionnaire) to provide your contact details.**

If you would like to participate, please complete this questionnaire by 28th October 2022.

**Proceeding to the next page will be taken as confirming your consent to participate in this consultation.**

*If you do not wish to take part, simply ignore this questionnaire.*

# About you

## About you and your your type of expertise

*Your answers to the following questions will help us to describe the composition of the group of people who have responded. This will help us to show the variety of experience and expertise represented. Your responses will be kept confidential. The information will only be reported in aggregate, e.g. the proportions of men and women.*

Choose one: I am...

- Female
- Male
- Other
- I would prefer not to answer

Choose one: How old are you?

- 16-25
- 26-45
- 46-65
- 66-75
- 76-85
- 86 years or older
- I would prefer not to answer

We are interested in two groups of people for this consultation. One includes people who are currently engaged or involved in the design, construction, supply, or management of housing or in other housing-related fields. We call this 'experts by profession'. The other

group are people who are aged 50 years or older and own, rent, or occupy their own homes. They can live alone or with other people. We call these 'experts by experience'. Choose one: I am an...

- ◯ 'Expert by profession', someone who is engaged or involved in a housing-related field
- ◯ 'Expert by experience', a person who owns, rents, or occupies a home

# About you and your housing-related professional expertise

The questions in this section are for  'experts by profession', i.e. people engaged or involved in different housing-related fields. If this does not describe you, please click on the 'Previous' button at the foot of the page to go back to the previous page and select the 'expert by experience' option on the final question.

The answers to these questions will help us to describe the breadth of professional experience represented. Your responses will be kept confidential. You do not have to answer any question that you do not want to. All questions contain an 'I would prefer not to answer' option.

Choose one: The housing-related area that I am involved in is... ✱ *Required*

- ○ Design-related (e.g. Architect, Planner, Developer)
- ○ Construction-related (e.g. Surveyor, House-builder)
- ○ Housing supply/management-related (e.g. Landlord, Housing officer, Letting agent)
- ○ Occupier support-related (e.g. Housing options officer, Resident engagement officer, Support worker)
- ○ Housing sales, marketing and/or communications-related (e.g. Sales person, Marketing officer, Estate agent)
- ○ Policy-related (e.g. Housing policy officer, Housing advocate/activist, Lobbyist)
- ○ Other
- ○ I would prefer not to answer

If you selected Other, please specify:

[ ]

Choose one: The main organisation though which I am involved in a housing-related field is... ✱ *Required*

- Private or for-profit organisation
- Public sector (central or local government) organisation
- Third sector or not-for-profit organisation
- Other
- I would prefer not to answer

If you selected Other, please specify:

Choose one: My role in this organisation is... ✱ *Required*

- Strategic (e.g. director/board member/trustee)
- Management and/or administration (primarily internal-facing role, e.g. service manager, HR professional)
- Operational (primarily external-facing role, e.g. support worker)
- Other
- I would prefer not to answer

If you selected Other, please specify:

Choose one: In total, including paid and/or voluntary work in this and other organisations, I have been involved in this housing-related field for... ✱ *Required*

- 0-5 years
- 6-10 years
- 11-15 years
- 16 or more years

- [ ] I would prefer not to answer

# About you and your current home

The questions in this part of the questionnaire are for 'experts by experience', i.e. people who are older home owners or occupiers. If this does not describe you, please click on the 'Previous' button at the foot of the page to go back to the previous page and select the 'expert by profession' option on the final question.

The answers to these questions will help us to know whether we have included people with different personal circumstances.

Any information that you give us will be treated as confidential. You do not have to answer any question that you do not want to. All questions in this section contain an 'I would prefer not to answer' option.

## About your current health and memory

The next four questions ask about your current health and your memory. Your answers will help us to describe the health circumstances of people who have responded.

How is your health in general? Is it…

- Very good
- Good
- Fair
- Bad
- Very bad
- I would prefer not to answer

Do you have any longstanding illness or health problem? (By longstanding we mean illnesses or health problems which have lasted, or are expected to last, for 6 months or more)

- Yes

- No
- I would prefer not to answer

For at least the past 6 months, to what extent have you been limited in activities that you would usually do because of a health problem? Would you say you have been…

- Severely limited
- Slightly limited
- Not limited at all
- I would prefer not to answer

How much difficulty do you have remembering important things?

- No difficulty
- A little difficulty
- A lot of difficulty
- Unable to remember important things
- I would prefer not to answer

## About your current housing situation

The following questions ask about your current home and household. The answers to these questions will help us to describe the housing situations and experiences of people who responded.

Choose one: my home is...

- Rented from a council or housing association

- Rented from a private landlord
- Owned by me with a mortgage
- Owned by me outright
- Other
- I would prefer not to answer

If you selected Other, please specify:

Choose one: My home is...

- A detached house or bungalow
- A semi-detached house or bungalow
- A terraced or end-of-terrace house or bungalow
- A flat, maisonette or apartment in a purpose-built block of flats or tenement
- A flat or apartment that is part of a converted or shared house, including bedsits
- A flat or apartment that is part of another converted building (e.g. a converted mill or former factory building)
- A flat or apartment that is part of a commercial building or building with another purpose (e.g. a flat above a shop, a room or rooms in a hotel)
- A mobile or temporary structure (e.g. a campervan, houseboat, or static caravan)
- Other
- I would prefer not to answer

If you selected Other, please specify:

Tick all that apply: in terms of who lives in my home...

- ☐ I live alone
- ☐ My spouse or partner lives with me
- ☐ One or more of my and/or my spouse or partner's children lives with me
- ☐ One or more of my and/or my spouse or partner's parents lives with me
- ☐ One or more of my and/or my spouse or partner's other relatives (e.g. brother or sister, grandchildren) lives with me
- ☐ One or more other person who is not related to me or to my spouse or partner lives with me
- ☐ I would prefer not to answer

# Your perceptions of the possible benefits of housing which can support healthy cognitive ageing

The DesHCA project aims 'to develop evidenced, scalable new and retro-fitted housing design which can support healthy cognitive ageing for rich and poor alike, delivering added years of healthier, independent ‘ageing in the right place’ for all'.

We want to know your thoughts on what kinds of benefits or positive changes this type of housing could lead to. In the following questions we ask you to think about possible benefits or positive changes for three groups:

a) people who live in housing which can support healthy cognitive ageing;

b) neighbourhoods and communities that have housing which can support healthy cognitive ageing;

c) individuals and organisations which design, build, supply, and manage this housing which can support healthy cognitive ageing.

There are no right or wrong answers. This type of housing is **not** currently widely available. We are asking you to consider what the benefits or positive changes could be if it were available.

## Your perception of the possible benefits for people who live in housing which can support healthy cognitive ageing

The DesHCA project is looking at supportive design and/or adaptation across the home as a whole. The following questions ask you to think about how housing, which supports healthy cognitive ageing, might have positive benefits for what activities people can do in their homes and how they feel about their homes.

Housing which supports healthy cognitive ageing might help people do different kinds of activities within the home. **What kinds of activities do you think homes could help with?** You might think about what activities a future, older, version of yourself would want to do. Activities could be specific, e.g. taking a bath, or more general, e.g. spending time in the garden. *Briefly describe activities which you think homes should help people do in the text box below.*

Housing which supports healthy cognitive ageing might positively influence how people feel. **How do you think 'home' should make a person feel?**
You might consider what you would want a future, older, version of yourself to feel about their home.
*Please type your thoughts into the text box below.*

## Your perceptions of the possible benefits to neighbourhoods and communities

Housing which supports healthy cognitive ageing might benefit local neighbourhoods or communities. **In an ideal world, how would this housing benefit local neighbourhoods or communities?** You might think about how the housing might contribute to the kind of neighbourhood or community that you would like to live in later in your life. *Please type your thoughts in the text box below.*

## Your perceptions of the possible benefits for people who design, build, supply, and/or manage housing

The questions which follow ask you to think about benefits for different groups of people or organisations who work in design, construction, supply, or management of housing.

There are no right or wrong answers. If you do not feel able to make suggestions in some cases, please just leave that text box empty.

**What benefits might there be for those involved in the design and development (e.g. architects, planners, developers) of housing which supports healthy cognitive ageing?** You might consider benefits for those directly involved in design and benefits to the wider sectors with which they are associated. *Please briefly describe any benefits or positive changes that you think involvement in the design of housing could bring in the text box below.*

**CONSTRUCTION. What benefits might there be for individuals, organisations or bodies with roles in housing construction (e.g. building and construction professionals, building control) involved in the development of housing which supports healthy cognitive ageing?** You might consider benefits for those directly involved in construction and for the wider sectors with which they are associated. *Please briefly describe any benefits that you think involvement in the construction of housing which supports healthy cognitive ageing could bring in the text box below.*

**SUPPLY. What benefits might there be for individuals, organisations or bodies involved in supplying housing (e.g. house-builders, housing associations, local authorities) which supports healthy cognitive ageing?** You might consider benefits for those directly involved and for the wider sectors with which they are associated. *Please briefly describe any benefits that you think involvement in the supply of housing could bring in the text box below.*

**MANAGEMENT. What possible benefits or positive changes might there be for individuals, organisations or bodies that manage housing (e.g. housing associations, local authorities, landlords) which supports healthy cognitive ageing?** You might consider benefits for those directly involved and for the wider sectors with which they are associated. *Please briefly describe benefits that you think involvement in the management of housing which supports healthy cognitive ageing could bring in the text box below.*

# Your thoughts on the questions we have asked

Please use this space to let us know of any additional thoughts, comments or feedback that you might have in relation to the questions we have asked

# Final page

## Final Section

Thank you for taking the time to participate in this consultation.

When we have collected all the responses, we will use them to examine agreements and disagreements about what is important for different groups. Then we will test these results. We will do this using an 'eDelphi' approach, which means we will send three short questionnaires at monthly intervals (November 2022-January 2023).

**If you would like to take part in this future exercise, please enter your email address below.**
