# Supplementary material for: Outcomes of home design to support healthy cognitive ageing: modified e-Delphi exercise with older people and housing-related professionals
Source: BMC Geriatr. 2024 Jun 24;24:546. doi: 10.1186/s12877-024-05085-z (PMC11194886; doi:10.1186/s12877-024-05085-z)
Supplement: Supplementary file 3 — Supplementary Material 3 [file 12877_2024_5085_MOESM3_ESM.docx]

## Welcome to Round 1 of the DesHCA Project's eDelphi process!

**About the project**

The Designing Homes for Healthy Cognitive Ageing (DesHCA) project is a large, UKRI funded research project based at the University of Stirling that explores the different ways that peoples homes can be designed, built, or adapted to better support them as they age. Our research involves working alongside people from different walks of life. industry backgrounds, and professional settings to examine how adapting existing homes could help older people in the here and now, as we work alongside architects and builders to design more supportive homes for the future.

## About this eDelphi survey

This survey has been designed to help us learn more about what people think about when we talk about designing supportive homes, what they'd like those homes to look like, and what they'd like the creation of more supportive homes to mean- for themselves as individuals, their communities, and for the industries and professionals working to design, build, manage and adapt homes across the UK. It was built using the responses to an earlier, exploratory survey where we asked volunteers to tell us about how they thought different groups might benefit from the creation of more supportive housing in the UK.

Taking part in the eDelphi process will involve filling out three surveys over the next three months, starting with this one. We expect each survey to take around 15 minutes to complete, and we will give you an insight into the answers we received during each round so you can learn more about how people from others backgrounds, age groups, or professions answered the questions, and compare their answers to your o n.

We ill use your ans ers alonQside those of other volunteers to develop a set of outcomes, or Qoals, that e ill report on hen researchinQ or talkinQ about supportive home desiQn. This ill help us make sure e're ans 1erinQthe questions that really matter, rather han focusing exclusively on hat academic research says should matter.

## What is Healthy Cognitive Ageing?

We use he ords 'healthy cognitive ageing' as an umbrella term to talk about he many different health conditions or diagnoses that might impact ho a person's brain • orks as they age. Designing for healthy cognitive ageing doesn't just mean designing homes o support people living ith dementia. Parkinson's, or o her specific health conditions, but designing homes that are more inclusive, accessible, and supportive for anyone

ho might begin to experience changes in ho their brain 1orks as they get older.

Designing homes for healthy cognitive ageing means designing homes hat support people to do the things that matter o them throughout their life, hether they4re hiking Munro's in their 90s, recovering from surgery in their 70s, or living ith early onset dementia in heir 50s.

## Who can take part in the eDelphi?

These eDelphi surveys are open to people living in the UK rho are g) over 55 orb) working in an area involved in designing. buildinQ. maintaining. adaptinQ._Q[_Qroviding housinQ in the UK, or c) both. Its important tha e Qather as many insiQhts from people within these Qroups as possible so e can better understand;the issues and hopes around supportive housinQ o key perspectives: the professionals involved in creating more supportive homes, and the people who might choose to live in them.

**When will the other surveys go live?**

As the firs survey in the eDelphi process. this survey ill remain open unrn12pm midday on Friday 13th January.

links to the second survey will t:hen be emailed to you on the *eek beginning Monday the 16th of January·.*it:h;the survey clos,ing at 11:59pm on Sunday 5th of February. lin'ks to the final survey in the panel·.ill then be emailed to you on *the week beginning*

*the 13th of February,* and·,mclose at 11:59pm Sunday the St1h o March.

**Further lnforimation**

n is :important to note that your participation t!he eDelphi process 1is entire:iv voluntary: you do not have to take part if you do not wish to, and you can ·•it,hdra ft"om the process at any t,ime. ithout providing a reason.

All ans ers collected through the eoe:lp:hi surveys will be anonymised, meaning •• e .••.m never reveal your details o tihose outside t1he s udy. While the survey does ask for some details about you (gender,!life experiences) these are only intended to p:rov1ide context for the ans iers you give..and lhetp us understand any patterns . e notice in the data.

Information gathered by the eDelphi ill be used to inform other researoh,ao ivities

,ithin tihe DesHCA project (1inoluding the design of our VR home models and our Serious Game), and may be reported 1in other.·ays suc:h as academic papers, policy briefings, presentations. and biogs. We may use a summary of the data collected during

- he eDelph1i surveys to do this, including picking ou1 specific quotes or opinions o high'ligh :key messages- but these wm always be anonymised.

We hope that you feel able to comp:lete t•his survey- ho ever *if* at any time you •,ish to stop, you may do so by clos·ing the bro ser . indo . If you exi the survey before

ans· erin the fina'I question, your responses , ill not be inc,tuded ·n the pfoject. If you complete the survey but chanQe your mi11d and ·• ish to . itlldra you, responses, you may do so by emailing Catherine Pemble at [catherine.pemb.le@stir.ac.uk](mailto:catherine.pemb.le@stir.ac.uk) ithin 2 wee'ks of completing ttie survey and prov1iding yourname.

n you experience problems accessing this survey online and oud like to complete the survey over the telephone 1instead. then p1ease email [catherine.pemble@stir.ac.uk](mailto:catherine.pemble@stir.ac.uk) to arrange a tiime to do thiis. If you · ould like Jurther informatiion abou th1is project, you can email the Principal Investigator, Professor AJison Bo.es, at [a.m.bo.1es@stir.ac.uk.](mailto:a.m.bo.1es@stir.ac.uk) If you:have concerns about this survey, or the conduct of this project, them please

contact Professor Liz IForbat, Deputy Dean of the Faculty of Social Sc,iences, ,by email at e:lizabe [hJor.bat1@stir.ac.uk.](mailto:hJor.bat1@stir.ac.uk)

'If you understand ,he information above, and are happy to complete the survey, p.lease ans er the quesuons below. and Olick 1Next1 to proceed to the next screen.

0 I am over ,the age of 55 and living in the UK

0 I am over the age ,of 55, liv,ing in the UK, and have a pro essional background in an area related ,to housing (e.g. design, construction, adaptation,, prov,ision, management etc.)

0 I am under the age of 55, living in the UK, and hav,e a 1professional background in an area relate,dt,o housing (e.g. design, construction, adaptation, prov,ision, management

·etc.)

Taking part in this activity wiU invorve answering three surveys in tota11: th,e one you

ar,e reading today, a follow up survey in January 2023, and a final survey in February 2023.

Please enter the email address you \i ould like us to use to contact you below.

What is your name?

Ho old are you?

How woul:d you describe your gender?

We'd like to kno. a little more about your life experiences. Please se1lect the options below hich apply to you.

I am [living i,ith, or have lived ith, a health condtion that affects my hearing, sight, or o her se ses

D D

I am living ith or have lived . ith, a health condtion that affects how easily I can move around

D D

I am living Yith, or have lived ith, a health condtion that affects my thinking, memory, or cogn 10n

D D

D None of , hese apply to me D

I have .• orked with people living with a health condtion that affected their hearing, sight, or ,other se ses as part of my professional role

I have • orked with people living ith a health condtion that affected ho easily they could move around as part of my professional role

I have orked with people living · ith a health condtion that affected their thinking, memory, or cognition as part .of my pmfessional role

I iprefer not to ans er

Hm does your professional ba,ckground relate to hous·ing?

# The Designing Homes for Healthy Cognitive Ageing (DesHCA) Project is a multi-year UKRI funded project that focuses on understanding the different ays that a home could be designed, modified, or adapted to better support people as they age by planning and accounting for potential changes in physical ability, sensory impairment, or difficulties v ith thinking, memory, or cognition.

Volunteers who responded to our first survey talked about hat they would like a supportive home to be. The options below ere dra1J n from their responses.

Please select what you think are the **10 most important things that a supportive home should be.**

# Please note e are not asking you to rank these options in order of their importance, just highlight the ten options that are the most important to you.

Designed to support people i h sensory

0 impairments (such as loss of vision or hearing loss)

Designed to support people vho ave

Built in an area tha focuses on providing

**0** homes and services to older people

Built in an area here people from

issues • i h eir mobili (this may include differen ages or community groups live

0 using a heelchair, a alker, or o er alking .aid, or people ho have eal h

conditions that make it difficult to move, such as arthritis)

Designed to support people i h cognitive change (this may include people ho live

D ith conditions like dementia and

Parkinson's, as • ell as some stroke survivors and peop1e ith learning disabilities etc)

**O** ogether

Designed to make it easy to install telecare systems or other caring technology (such

D as communi alarms, pendants and fall

se sors)

Built in a place that makes it easy to Equipped i h Smart Home technology

**0** access to community spaces, shops, and **0**

services by bus, trai , or car

Built in a place that makes it easy to

**0** access community spaces, shops, and

services by walking

Easy to keep arm, or cool, as needed

**D**

Designed to make it eas·er for people to Designed ith e ra space i mind (e.g.

**0** install adaptations if they need them (such **O** should not feel like the 'smallest possible' as stair lifts, hoists, or grab bars) home)

Designed to look as beautiful, modern, or Affordable home-like as other houses in the area (e.g.

**0** sho Id no look' like a home designed for **0**

an older person, or someone living i h health issues}

Designed to be flexible, so the people ho A haven or sanctuary for the people that

0 live there can easily change things to suit **O** live there heir prefere ces and needs

**0** Fitted ith applia ces and utilities hat are **0** Desirable; somewhere people wan o live

easy to use and understand

**D** Designed to redure the risk of falls and the fear of falling

no matter their age

A home that supports healthy cognitive ageing would be desig ed to make i as easy as possible for the people living there to keep doing the activi ies that are important to them as they get older.

The options belo are ased on he ans ers given by volunteers in our firs questionnaire,

" here e asked people about the kinds of activities that should be made easier by living in a supportive home.

Please select ha you think are the **10 most important activities** that should be supported by a home designed to promote heal hy cognitive ageing from the options below.

0 Spendi g time outside

0 Gardening

0 Exercising

0 Staying physically active

0 Listening to music

0 Having pets & animal compa ions

0 Using a car or mobility scooter

0 Staying independent

0 Have a normal ilife as I get older

0 Getting ou and about in the communi

O Crafting hobbies (painting, knitting, model O Socialising ith family & f·ends building etc)

0 Reading

0 Playing games & boardgames

0 Keep doing the ac ivities I enjoy

0 Ba . ing, showering & staying clean

0 Having a bath

0 Going to he toilet

0 Getting dressed

0 Having lunch or dinner with visitors

0 Prepa g food & coo• ng meals

Using the computer/tablet or other technology

D

0 Housework & keeping he house clean

0 Making repairs & maintaining the house

0 S aying safe

0 Doing he laundry

Living in a supportive home should be a positive experience. e asked volunteers from our first questionnaire about ho a home designed to support healthy cognitive ageing should make people feel. The options belo are based on their ans ers.

Please select what you think are the **10 most important things people should feel when living in a home that supports healthy cognitive ageing** from the options belo

| **D** | Safe or Secure | **D** | I dependent |
| --- | --- | --- | --- |
| **D** | Happy or Content | **D** | Valued |
| **D** | Relaxed | **D** | Calm |

Like their home reflects their preferences Part of their community or style

**D D**

| **D** | Able to change heir home to suit their needs | **D** | Warm |
| --- | --- | --- | --- |
| **D** | Sociable, or connected with others | **D** | Like their home is a private space |
| **D** | Protected | **D** | Financially secure |
| **D** | Comfortable | **D** | Fulfilled |
| **D** | Supported or Enabled | **D** | Like their home is a space to have fun in |
| **D** | In control of their home |  |  |

Building housing that supports older people to live in the home of their choosing for longer may have positive outcomes for our neighbourhoods and communities.

We asked volunteers from our first survey about the different \ ays they thought building more supportive housing might improve their local community.

P,lease select what you think are the **top 10 most important outcomes for the community** from the options below.

| **0**  **O** | More community engagement  More conne ·on between members of the | 0  **O** | Communities become more supportive  Development of more ac ivities within the |
| --- | --- | --- | --- |
|  | community |  | community |
| **O** | More intergenerational activities a d spaces | **0** | Community keeps a connec·on to its history |
| **0** | Residents stay in their community longer | **0** | Community spaces are better maintained |
| **D** | Communities become more indusive | **0** | Reduced pressure o public services |
| **D** | Communities become more physically accessible | **0** | More opportunities for employment |
| 0 | Local services are used more often | 0 | Communities become more focused towards older people |
| **O** | More demand for shops and businesses | O | Older members of the community can provide mento• ng for younger people |
| **0** | More demand for community spaces | **0** | Improvements to transportation |
| **O** | More demand for outdoor or green spaces | **O** | Older and younger people can learn from each other |

It's important that we consider the different\ ays that building more supportive homes for older people might impact different parts of our society.

We asked volunteers in survey one about ho they thought those involved ith designing and developing new buildings (such as architects, designers, and planners) might benefit from creating more housing that supports healthy cognitive ageing. The options belo are drawn from their answers.

Please select what you think are the **top 10 benefits for those working in design and development** from the options belo

**0 U** housing stock is improved

O Professionals learn more abou people need and • ant

**0** More supportive housing creates a better future

hat older D Older people can live for longer in he homes and communities of their choosing

| 0 | Professionals learn more about the principles of supportive design, or | **0** | Supportive houses reduce pressure on  pub ic services |
| --- | --- | --- | --- |
|  | designing for older people |  |  |
| **0** | Designs for all houses improve | **0** | Supportive houses improve oommuni·es |
| **D** | Professionals gain more job satisfaction | **D** | Supportive houses reduce care home admission |
| **0** | Supportive houses open up ne markets, customers, or ew opportunities for profit | **0** | Professionals and companies that build supportive houses gain pos·ve recognition |
|  |  |  | and PR |

Professionals t at build supportive houses

**O** are given more opportunities or contracts **O**

Professionals learn more about \ hat people living with conditions that lead to cognitive change (such as dementia, Parkinson's or a stroke) need or ant

It's important that e co sider the different ays that building more supportive homes for older people might impact different parts of our society.

We asked volunteers in our first survey about ho they thought designing more supportive homes might benefit professionals working in construction (such as builders and building control experts). The follo ing options are dra n from their ans\ ers.

Please select what you think are the **top 10 benefits for professionals working in construction** from the options belo

Professionals learn more about the

**0** principles of supportive design, or

designing for older people

D Professionals gain more job satisfaction

Supportive houses reduce pressure on

**0** public services

D Older people can live for longer in he homes and comm nifes of their choosing

Supportive houses open up ne markets, customers, or ew opportunities for profit

**D D**

Professionals learn more about hat older people need and ant

**D** D

**D** UK housing stock is improved **D**

Supportive houses reduce care home admission

**D** D

More opportunities for employment

**D** D

Professionals learn more abo t "lhat people living with conditions that lead o cognitive change (such as dementia, Parkinson's or a stroke) need or want

**D**

S pportive homes will be more sustainable

More supportive housing creates a better future

Guidance and regulations will improve

Professionals that build supportive houses are given more opportuni·es or contracts

Professionals and companies that build suppo ·ve houses gain positive recognition and PR

It's important that e consider the different ways that build1ing more supportive homes for older people might impact different parts of our sodety.

In our previous survey e asked volunteers to suggest how build1in.g homes that support healt,y cognitiv·e ageing might benefit organisations and auti orities who supp'ly hous,ing (such as house builders, housi g associations, and local authoriti,es). The options below are drawn from thefr answ,ers.

Please se·l,ect what you think ar,e the **top 10 benefits for those who supply housing** from the options belo

| **0** | **UK** housing stock·s improv,ed | O | P1rofossiona'ls improve their practice overall |
| --- | --- | --- | --- |
| O | Support1ive houses open up ne | markets, O | Professionals gain more job satisfaction |

cust,omers, or new opportunities for profit

**O** Su:pportiv.e houses r,educe pressure on O More opportunities for .employment public services

**O** Older people ca live for longer in the **O**

homes and communit,ies of their choosing

Profossionals learn more about what older

Communities become more inclusive

Pirofessiona'ls and companies that supply

**0** people need and ant

**0** suppo ·ve houses gain pos· ive recognifon and PR

**O** P.rofossionals that bu1iad support,ive houses O Communities become bet er and more

are giv,en more oppo.rtunitries or contracts supportive

□ ore suppo ·v,e housing cr,eates a better O Supportive homes are built to be mor,e futur,e sus ainable

Professionals learn more abo t hat

**O** people living with cond,itions that ,lead o

cognitive dhange (such as dementia, Parkinson's or a stroke) need or want

It's important that we consider the different\ ays that bu:ildin,gmore supportive homes for older people might impact different parts of our sodety.

In our last survey we asked about how building more cognitive supportive hous·ing might benefit the 1individuals, organisations, or bod.ies that mana,ge housing (such as housing associations, 1local authoriti·es, or landlords). The options below· •ere dra n from their ans ers. *This is the last question of this type.*

Please select what you think might be the **to,p 10 benefits for those who m.anage housing** from the 01ptions belo· .

| 0  **D** | Support1ive houses are easier to manage  SupportIive houses r•educe ,pressure on | 0  O | Professionals improv,e their practice overall  More supportive housing crea,tes a better |
| --- | --- | --- | --- |
|  | pub1jc services |  | future |
| 0 | Suppo.rt,ive houses open up ne markets, customers, or new opportunities for profit | 0 | More opportunities for employment |
| D | Professiona'ls leam more abou • hat older peop:le need and an,t | 0 | Housing stock bec·omes m,ore flexib'le and suits more people's needs |
| O | Older people can .liv,e for !longer in the homes and communities of their dhoosing | D | Housing stock requires 'less adapta ion in the future |
| 0 | Their housing stock is improved | 0 | Professionals gain more job satisfaction |

Support,ive houses make it easier to

0 provide residents . ith su;pport

Professionals ·learn more about what

D people livi,ng with conditions tha lead to

oognifve ohange (such as dementia,, Parkinson's or a strok,e) need or· ant

Volunteers in our last survey suggested that building more supportive housing might have a positive effect on health and social care services (such as social workers, GPs, occupational therapists, and emergency services). The options belo were drawn from their suggestions.

## Please drag and drop the the options below so that the largest, or most important outcome is at the top, and the smallest or least important outcome is at the bottom.

Supportive design may make it easier for health and social care professionals to adapt omes for people's needs later.

Supportive homes may make it easier to provide care o someone liv,ing at home

Supportive homes may reduce demand for health a d social care serv·ces by supporting independence a d improving people's mental health and wellbeing

Supportive homes may reduce the risk of hospital admission

Supportive homes may reduce the risk of people moving to a care home if they do not

- ish to

Building more supportive homes may reduce he number of people living in housing hat does not suit them

Supportive homes may reduce the risk of delayed release from hospital

Supportive design may make it easier for people ho are living ith dementia or cognitive change to live in the home of heir choosing for longer
