# Supplementary material for: Outcomes of home design to support healthy cognitive ageing: modified e-Delphi exercise with older people and housing-related professionals
Source: BMC Geriatr. 2024 Jun 24;24:546. doi: 10.1186/s12877-024-05085-z (PMC11194886; doi:10.1186/s12877-024-05085-z)
Supplement: Supplementary file 4 — Supplementary Material 4 [file 12877_2024_5085_MOESM4_ESM.pdf]

Welcome to Round 1 of the DesHCA Project's eDelphi process!

About the project
The Designing Homes for Healthy Cognitive Ageing (DesHCA) project is a large, UKRI funded research project based at the University of Stirling that explores the different ways that peoples homes can be designed, built, or adapted to better support them as they age. Our research involves working alongside people from different walks of life, industry backgrounds, and professional settings to examine how adapting existing homes could help older people in the here and now, as we work alongside architects and builders to design more supportive homes for the future.

About this eDelphi survey
This survey has been designed to help us learn more about what people think about when we talk about designing supportive homes, what they'd like those homes to look like, and what they'd like the creation of more supportive homes to mean- for themselves as individuals, their communities, and for the industries and professionals working to design, build, manage and adapt homes across the UK. It was built using the responses to an earlier, exploratory survey where we asked volunteers to tell us about how they thought different groups might benefit from the creation of more supportive housing in the UK.

Taking part in the eDelphi process will involve filling out three surveys over the next three months, starting with this one. We expect each survey to take around 15 minutes to complete, and we will give you an insight into the answers we received during each round so you can learn more about how people from others backgrounds, age groups, or professions answered the questions, and compare their answers to your own.

We will use your answers alongside those of other volunteers to develop a set of outcomes, or goals, that we will report on when researching or talking about supportive home design. This will help us make sure we're answering the questions that really matter, rather than focusing exclusively on what academic research says should matter.

What is Healthy Cognitive Ageing?
We use the words 'healthy cognitive ageing' as an umbrella term to talk about the many different health conditions or diagnoses that might impact how a person's brain works as they age. Designing for healthy cognitive ageing doesn't just mean designing homes to support people living with dementia, Parkinson's, or other specific health conditions, but designing homes that are more inclusive, accessible, and supportive for anyone who might begin to experience changes in how their brain works as they get older.

Designing homes for healthy cognitive ageing means designing homes that support people to do the things that matter to them throughout their life, whether they're hiking Munro's in their 90s, recovering from surgery in their 70s, or living with early onset dementia in their 50s.

Who can take part in the eDelphi?
These eDelphi surveys are open to people living in the UK who are a) over 55 or b) working in an area involved in designing, building, maintaining, adapting, or providing housing in the UK, or c) both. Its important that we gather as many insights from people within these groups as possible so we can better understand the issues and hopes around supportive housing two key perspectives: the professionals involved in creating more supportive homes, and the people who might choose to live in them.

When will the other surveys go live?

As the first survey in the eDelphi process, this survey will remain open until 12pm midday on Friday 13th January.
Links to the second survey will then be emailed to you on the *week beginning Monday the 16th of January* with the survey closing at 11:59pm on Sunday 5th of February.
Links to the final survey in the panel will then be emailed to you on *the week beginning the 13th of February*, and will close at 11:59pm Sunday the 5th of March.

Further Information

It is important to note that your participation the eDelphi process is entirely voluntary: you do not have to take part if you do not wish to, and you can withdraw from the process at any time without providing a reason.

All answers collected through the eDelphi surveys will be anonymised, meaning we will never reveal your details to those outside the study. While the survey does ask for some details about you (gender, life experiences) these are only intended to provide context for the answers you give, and help us understand any patterns we notice in the data.

Information gathered by the eDelphi will be used to inform other research activities within the DesHCA project (including the design of our VR home models and our Serious Game), and may be reported in other ways such as academic papers, policy briefings, presentations, and blogs. We may use a summary of the data collected during the eDelphi surveys to do this, including picking out specific quotes or opinions to highlight key messages- but these will always be anonymised.

We hope that you feel able to complete this survey- however if at any time you wish to stop, you may do so by closing the browser window. If you exit the survey before answering the final question, your responses will not be included in the project. If you complete the survey but change your mind and wish to withdraw your responses, you may do so by emailing Catherine Pemble at catherine.pemble@stir.ac.uk within 2 weeks of completing the survey and providing your name.

If you experience problems accessing this survey online and would like to complete the survey over the telephone instead, then please email catherine.pemble@stir.ac.uk to arrange a time to do this. If you would like further information about this project, you can email the Principal Investigator, Professor Alison Bowes, at a.m.bowes@stir.ac.uk. If you have concerns about this survey, or the conduct of this project, them please contact Professor Liz Forbat, Deputy Dean of the Faculty of Social Sciences, by email at elizabeth.forbat1@stir.ac.uk.

If you understand the information above, and are happy to complete the survey, please answer the questions below, and click 'Next' to proceed to the next screen.

- ○ I am over the age of 55 and living in the UK
- ○ I am over the age of 55, living in the UK, and have a professional background in an area related to housing (e.g. design, construction, adaptation, provision, management etc.)
- ○ I am under the age of 55, living in the UK, and have a professional background in an area related to housing (e.g. design, construction, adaptation, provision, management etc.)

Taking part in this activity will involve answering three surveys in total: the one you

are reading today, a follow up survey in January 2023, and a final survey in February 2023.

Please enter the email address you would like us to use to contact you below.

[ ]

What is your name?

[ ]

How old are you?

[ ]

How would you describe your gender?

[ ]

We'd like to know a little more about your life experiences. Please select the options below which apply to you.

- ☐ I am living with, or have lived with, a health condtion that affects my hearing, sight, or other senses
- ☐ I have worked with people living with a health condtion that affected their hearing, sight, or other senses as part of my professional role
- ☐ I am living with, or have lived with, a health condtion that affects how easily I can move around
- ☐ I have worked with people living with a health condtion that affected how easily they could move around as part of my professional role
- ☐ I am living with, or have lived with, a health condtion that affects my thinking, memory, or cognition
- ☐ I have worked with people living with a health condtion that affected their thinking, memory, or cognition as part of my professional role
- ☐ None of these apply to me
- ☐ I prefer not to answer

How does your professional background relate to housing?

[ ]

The Designing Homes for Healthy Cognitive Ageing (DesHCA) Project is a multi-year UKRI funded project that focuses on understanding the different ways that a home could be designed, modified, or adapted to better support people as they age by planning and accounting for potential changes in physical ability, sensory impairment, or difficulties with thinking, memory, or cognition.

Volunteers who responded to our first survey talked about what they would like a supportive home to be. The options below were drawn from their responses.

Please select what you think are the **10 most important things that a supportive home should be.**
Please note we are not asking you to rank these options in order of their importance, just highlight the ten options that are the most important to you.

- ☐ Designed to support people with sensory impairments (such as loss of vision or hearing loss)
- ☐ Designed to support people who have issues with their mobility (this may include using a wheelchair, a walker, or other walking aid, or people who have health conditions that make it difficult to move, such as arthritis)
- ☐ Designed to support people with cognitive change (this may include people who live with conditions like dementia and Parkinson's, as well as some stroke survivors and people with learning disabilities etc)
- ☐ Built in a place that makes it easy to access to community spaces, shops, and services by bus, train, or car
- ☐ Built in a place that makes it easy to access community spaces, shops, and services by walking
- ☐ Designed to make it easier for people to install adaptations if they need them (such as stair lifts, hoists, or grab bars)
- ☐ Designed to look as beautiful, modern, or home-like as other houses in the area (e.g. should not 'look' like a home designed for an older person, or someone living with health issues)
- ☐ Designed to be flexible, so the people who live there can easily change things to suit their preferences and needs
- ☐ Fitted with appliances and utilities that are easy to use and understand
- ☐ Designed to reduce the risk of falls and the fear of falling
- ☐ Built in an area that focuses on providing homes and services to older people
- ☐ Built in an area where people from different ages or community groups live together
- ☐ Designed to make it easy to install telecare systems or other caring technology (such as community alarms, pendants and fall sensors)
- ☐ Equipped with Smart Home technology
- ☐ Easy to keep warm, or cool, as needed
- ☐ Designed with extra space in mind (e.g. should not feel like the 'smallest possible' home)
- ☐ Affordable
- ☐ A haven or sanctuary for the people that live there
- ☐ Desirable; somewhere people want to live no matter their age

A home that supports healthy cognitive ageing would be designed to make it as easy as possible for the people living there to keep doing the activities that are important to them as they get older.

The options below are based on the answers given by volunteers in our first questionnaire, where we asked people about the kinds of activities that should be made easier by living in a supportive home.

Please select what you think are the **10 most important activities** that should be supported by a home designed to promote healthy cognitive ageing from the options below.

- ☐ Spending time outside
- ☐ Gardening
- ☐ Exercising
- ☐ Staying physically active
- ☐ Listening to music
- ☐ Crafting hobbies (painting, knitting, model building etc)
- ☐ Reading
- ☐ Playing games & boardgames
- ☐ Keep doing the activities I enjoy
- ☐ Bathing, showering & staying clean
- ☐ Having a bath
- ☐ Going to the toilet
- ☐ Getting dressed
- ☐ Having pets & animal companions
- ☐ Using a car or mobility scooter
- ☐ Staying independent
- ☐ Have a normal life as I get older
- ☐ Getting out and about in the community
- ☐ Socialising with family & friends
- ☐ Having lunch or dinner with visitors
- ☐ Preparing food & cooking meals
- ☐ Using the computer/tablet or other technology
- ☐ Housework & keeping the house clean
- ☐ Making repairs & maintaining the house
- ☐ Staying safe
- ☐ Doing the laundry

Living in a supportive home should be a positive experience. We asked volunteers from our first questionnaire about how a home designed to support healthy cognitive ageing should make people feel. The options below are based on their answers.

Please select what you think are the **10 most important things people should feel when living in a home that supports healthy cognitive ageing** from the options below.

- ☐ Safe or Secure
- ☐ Happy or Content
- ☐ Relaxed
- ☐ Like their home reflects their preferences or style
- ☐ Able to change their home to suit their needs
- ☐ Sociable, or connected with others
- ☐ Protected
- ☐ Comfortable
- ☐ Supported or Enabled
- ☐ In control of their home
- ☐ Independent
- ☐ Valued
- ☐ Calm
- ☐ Part of their community
- ☐ Warm
- ☐ Like their home is a private space
- ☐ Financially secure
- ☐ Fulfilled
- ☐ Like their home is a space to have fun in

Building housing that supports older people to live in the home of their choosing for longer may have positive outcomes for our neighbourhoods and communities.

We asked volunteers from our first survey about the different ways they thought building more supportive housing might improve their local community.

Please select what you think are the **top 10 most important outcomes for the community** from the options below.

- ☐ More community engagement
- ☐ More connection between members of the community
- ☐ More intergenerational activities and spaces
- ☐ Residents stay in their community longer
- ☐ Communities become more inclusive
- ☐ Communities become more physically accessible
- ☐ Local services are used more often
- ☐ More demand for shops and businesses
- ☐ More demand for community spaces
- ☐ More demand for outdoor or green spaces
- ☐ Communities become more supportive
- ☐ Development of more activities within the community
- ☐ Community keeps a connection to its history
- ☐ Community spaces are better maintained
- ☐ Reduced pressure on public services
- ☐ More opportunities for employment
- ☐ Communities become more focused towards older people
- ☐ Older members of the community can provide mentoring for younger people
- ☐ Improvements to transportation
- ☐ Older and younger people can learn from each other

It’s important that we consider the different ways that building more supportive homes for older people might impact different parts of our society.

We asked volunteers in survey one about how they thought those involved with designing and developing new buildings (such as architects, designers, and planners) might benefit from creating more housing that supports healthy cognitive ageing. The options below are drawn from their answers.

Please select what you think are the **top 10 benefits for those working in design and development** from the options below.

- ☐ UK housing stock is improved
- ☐ More supportive housing creates a better future
- ☐ Professionals learn more about what older people need and want
- ☐ Older people can live for longer in the homes and communities of their choosing
- ☐ Professionals learn more about the principles of supportive design, or designing for older people
- ☐ Supportive houses reduce pressure on public services
- ☐ Designs for all houses improve
- ☐ Supportive houses improve communities
- ☐ Professionals gain more job satisfaction
- ☐ Supportive houses reduce care home admission
- ☐ Supportive houses open up new markets, customers, or new opportunities for profit
- ☐ Professionals and companies that build supportive houses gain positive recognition and PR
- ☐ Professionals that build supportive houses are given more opportunities or contracts
- ☐ Professionals learn more about what people living with conditions that lead to cognitive change (such as dementia, Parkinson’s or a stroke) need or want

It’s important that we consider the different ways that building more supportive homes for older people might impact different parts of our society.

We asked volunteers in our first survey about how they thought designing more supportive homes might benefit professionals working in construction (such as builders and building control experts). The following options are drawn from their answers.

Please select what you think are the **top 10 benefits for professionals working in construction** from the options below.

- ☐ Professionals learn more about the principles of supportive design, or designing for older people
- ☐ Professionals gain more job satisfaction
- ☐ Supportive houses open up new markets, customers, or new opportunities for profit
- ☐ Professionals learn more about what older people need and want
- ☐ UK housing stock is improved
- ☐ Supportive houses reduce care home admission
- ☐ More opportunities for employment
- ☐ Professionals learn more about what people living with conditions that lead to cognitive change (such as dementia, Parkinson’s or a stroke) need or want
- ☐ Supportive houses reduce pressure on public services
- ☐ Older people can live for longer in the homes and communities of their choosing
- ☐ Supportive homes will be more sustainable
- ☐ More supportive housing creates a better future
- ☐ Guidance and regulations will improve
- ☐ Professionals that build supportive houses are given more opportunities or contracts
- ☐ Professionals and companies that build supportive houses gain positive recognition and PR

It’s important that we consider the different ways that building more supportive homes for older people might impact different parts of our society.

In our previous survey we asked volunteers to suggest how building homes that support healthy cognitive ageing might benefit organisations and authorities who supply housing (such as house builders, housing associations, and local authorities). The options below are drawn from their answers.

Please select what you think are the **top 10 benefits for those who supply housing** from the options below.

- ☐ UK housing stock is improved
- ☐ Supportive houses open up new markets, customers, or new opportunities for profit
- ☐ Supportive houses reduce pressure on public services
- ☐ Older people can live for longer in the homes and communities of their choosing
- ☐ Professionals learn more about what older people need and want
- ☐ Professionals that build supportive houses are given more opportunities or contracts
- ☐ More supportive housing creates a better future
- ☐ Professionals learn more about what people living with conditions that lead to cognitive change (such as dementia, Parkinson’s or a stroke) need or want
- ☐ Professionals improve their practice overall
- ☐ Professionals gain more job satisfaction
- ☐ More opportunities for employment
- ☐ Communities become more inclusive
- ☐ Professionals and companies that supply supportive houses gain positive recognition and PR
- ☐ Communities become better and more supportive
- ☐ Supportive homes are built to be more sustainable

It’s important that we consider the different ways that building more supportive homes for older people might impact different parts of our society.

In our last survey we asked about how building more cognitive supportive housing might benefit the individuals, organisations, or bodies that manage housing (such as housing associations, local authorities, or landlords). The options below were drawn from their answers. *This is the last question of this type.*

Please select what you think might be the **top 10 benefits for those who manage housing** from the options below.

- ☐ Supportive houses are easier to manage
- ☐ Supportive houses reduce pressure on public services
- ☐ Supportive houses open up new markets, customers, or new opportunities for profit
- ☐ Professionals learn more about what older people need and want
- ☐ Older people can live for longer in the homes and communities of their choosing
- ☐ Their housing stock is improved
- ☐ Supportive houses make it easier to provide residents with support
- ☐ Professionals improve their practice overall
- ☐ More supportive housing creates a better future
- ☐ More opportunities for employment
- ☐ Housing stock becomes more flexible and suits more people’s needs
- ☐ Housing stock requires less adaptation in the future
- ☐ Professionals gain more job satisfaction
- ☐ Professionals learn more about what people living with conditions that lead to cognitive change (such as dementia, Parkinson’s or a stroke) need or want

Volunteers in our last survey suggested that building more supportive housing might have a positive effect on health and social care services (such as social workers, GPs, occupational therapists, and emergency services). The options below were drawn from their suggestions.

Please drag and drop the the options below so that the largest, or most important outcome is at the top, and the smallest or least important outcome is at the bottom.

Supportive design may make it easier for health and social care professionals to adapt homes for people's needs later.

Supportive homes may make it easier to provide care to someone living at home

Supportive homes may reduce demand for health and social care services by supporting independence and improving people's mental health and wellbeing

Supportive homes may reduce the risk of hospital admission

Supportive homes may reduce the risk of people moving to a care home if they do not wish to

Building more supportive homes may reduce the number of people living in housing that does not suit them

Supportive homes may reduce the risk of delayed release from hospital

Supportive design may make it easier for people who are living with dementia or cognitive change to live in the home of their choosing for longer
