# Supplementary material for: Outcomes of home design to support healthy cognitive ageing: modified e-Delphi exercise with older people and housing-related professionals
Source: BMC Geriatr. 2024 Jun 24;24:546. doi: 10.1186/s12877-024-05085-z (PMC11194886; doi:10.1186/s12877-024-05085-z)
Supplement: Supplementary file 5 — Supplementary Material 5 [file 12877_2024_5085_MOESM5_ESM.pdf]

Intro

Welcome to Round 2 of the DesHCA Project's eDelphi process!

A Reminder About the Project

The Designing Homes for Healthy Cognitive Ageing (DesHCA) project is a large, UKRI funded research project based at the University of Stirling that explores the different ways that peoples homes can be designed, built, or adapted to better support them as they age. Our research involves working alongside people from different walks of life, industry backgrounds, and professional settings to examine how adapting existing homes could help older people in the here and now, as we work alongside architects and builders to design more supportive homes for the future.

About this eDelphi survey

The eDelphi survey has been designed to help us learn more about what people think about when we talk about designing supportive homes, what they'd like those homes to look like, and what they'd like the creation of more supportive homes to mean- for themselves as individuals, their communities, and for the industries and professionals working to design, build, manage and adapt homes across the UK.

This survey has been shaped by the answers provided by yourself and other volunteers during Round 1. Each section of this round will begin with an insight into the data collected during Round 1, followed by some questions that have been designed to help us gain a deeper understanding of why people made the choices that they did.

We recommend taking this survey on a computer or tablet device due to the interactive design of some of the questions included in this round. We expect this survey to take no more than **10 - 15 minutes to complete,** and will close to new responses at 10am on Monday 20th of February.

We expect to send out links to the final survey on the week beginning the **20th of March**.

Important information

Your participation the eDelphi process is entirely voluntary: you do not have to take part if you do not wish to, and you can withdraw from the process at any time without providing a reason.

All answers collected through the eDelphi surveys will be anonymised, meaning we will never reveal your details to those outside the study. While the survey does ask for some details about you these are only intended to provide context for the answers you give, and help us understand any patterns we notice in the data.

Information gathered by the eDelphi will be used to inform other research activities within the DesHCA project (including the design of our VR home models and our Serious Game), and may be reported in other ways such as academic papers, policy briefings, presentations, and blogs. We may use a summary of the data collected during the eDelphi surveys to do this, including picking out specific quotes or opinions to highlight key messages- but these will always be anonymised.

We hope that you feel able to complete this survey- however if at any time you wish to stop, you may do so by closing the browser window. If you exit the survey before answering the final question, your responses will not be included in the project. If you complete the survey but change your mind and wish to withdraw your responses, you may do so by emailing Catherine Pemble at catherine.pemble@stir.ac.uk within 2 weeks of completing the survey and providing your name.

If you experience problems accessing this survey online and would like to complete the survey over the telephone instead, then please email catherine.pemble@stir.ac.uk to arrange a time to do this. If you would like further information about this project, you can email the Principal Investigator, Professor Alison Bowes, at a.m.bowes@stir.ac.uk. If you have concerns about this survey, or the conduct of this project, them please contact Professor Liz Forbat, Deputy Dean of the Faculty of Social Sciences, by email at elizabeth.forbat1@stir.ac.uk.

If you understand the information above, and are happy to complete the next round of the eDelphi click 'Next' to proceed to the next screen.

What is your name?

Please re-enter your email address below.

Questions

Section 1 of 3

In the last round we asked people to select their 'top ten' options for what a home designed to suppoSrt someone as they get older should be. The graph below shows how volunteers voted last round, with items that are most 'important' (or most frequently chosen as part of the 'top ten') at the top, and less frequently chosen options at the bottom.

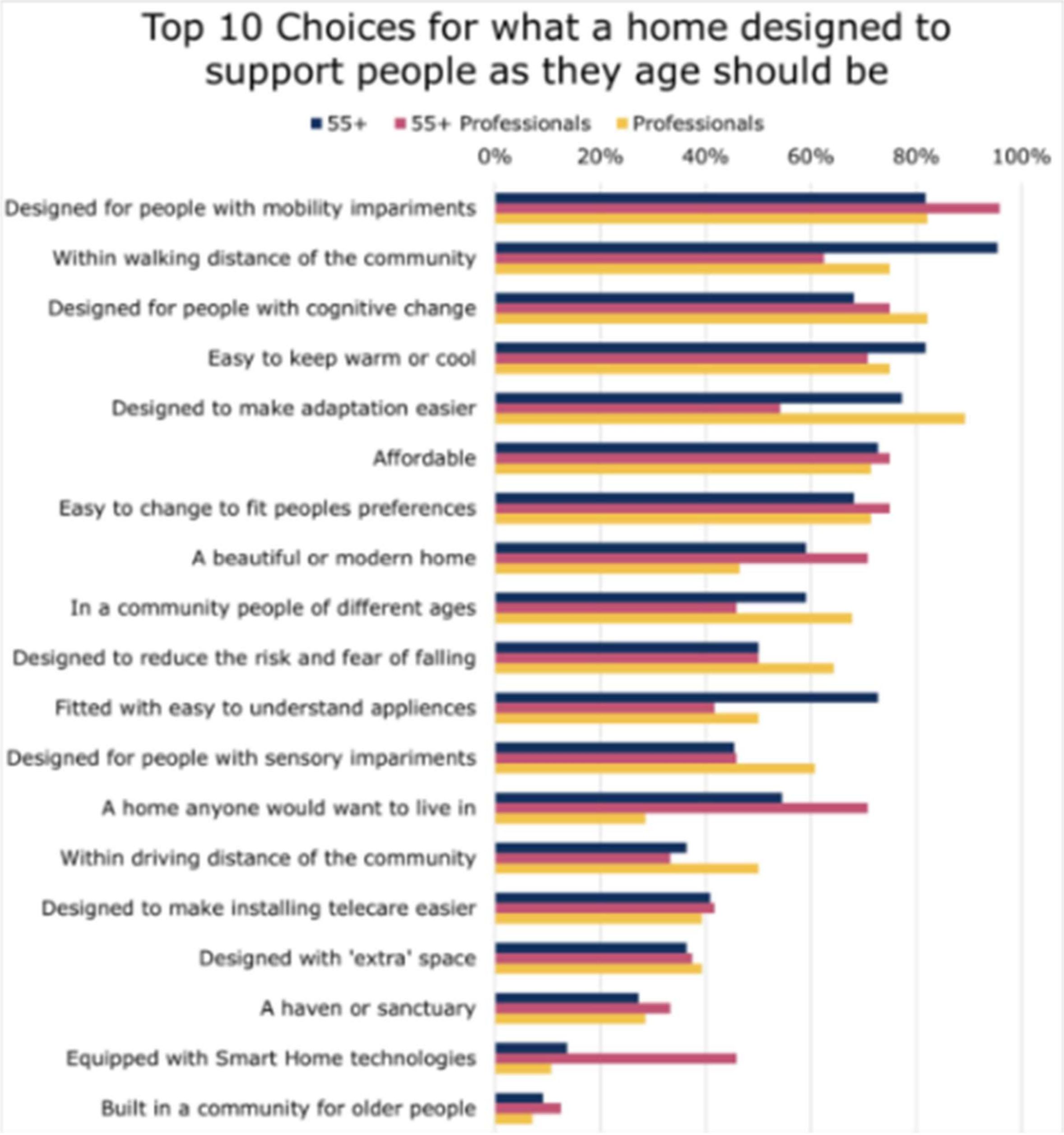

The following questions have been designed to help us understand these choices better.

What do you think about the pattern of answers shown in the graph above? How well does the ranking above match with your own thought about what a home designed to support people as they get older should 'be'?

Please read the statements below and select the option which best represents your opinion.

	Strongly disagree	Neither agree nor disagree	Somewhat agree	Strongly agree
It is possible to design or adapt a home to support someone who has difficulty with their hearing or sight	○	○	○	○
It is possible to design or adapt a home to support someone who has difficulty with their mobility or movement	○	○	○	○
It is possible to design or adapt a home to support someone who has difficulty with their memory, thinking, or cognition	○	○	○	○
Homes that are designed to support people as they get older should have Smart Home technology installed from the beginning	○	○	○	○
Homes that are designed to support people as they get older should make it easy to install remote care systems (like telehealth, or telecare) at a later date	○	○	○	○

Homes that are designed to support people as they get older should always be fitted with appliances that are easy to understand	○	○	○	○
Homes that are designed to support people as they get older should be built in places that make it easy to access the community by car	○	○	○	○
Homes that are designed to support people as they get older should be built in places that make it easy to access the community by public transport	○	○	○	○
Building homes that support people as they get older will lead to more demand for community spaces	○	○	○	○
Building homes that support people as they get older will lead to more demand for local shops and businesses	○	○	○	○
Building homes that support people as they get older will lead to people being more involved in their community	○	○	○	○

Section 2 of 3

In the last round we asked you to choose the 'top ten' things people should feel when they're living in a home that has been designed to support them as they get older. The graph below shows how our different groups of volunteers answered.

We've ranked these in order of 'most important' (most frequently chosen as part of the 'top 10') to least important.

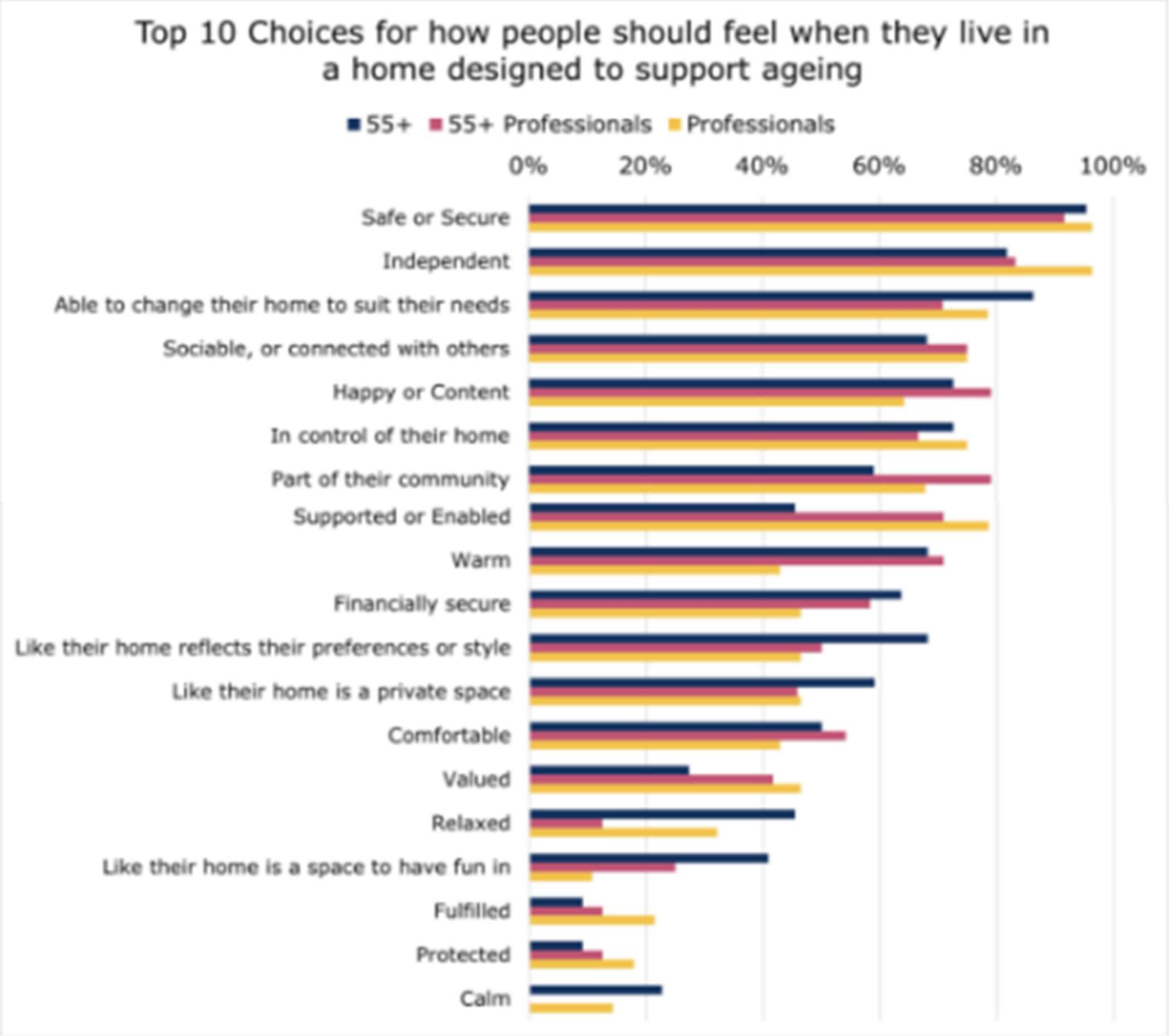

The next questions have been designed to help us understand these answers better.

What do you think about the pattern of answers shown in the graph above? How well does the ranking above match with your own thoughts about how someone should feel when living in a home that is designed with ageing in mind?

The next question is designed to help us understand this pattern by using the most commonly chosen options as 'sorting boxes' or umbrellas, to explore whether some of our original options might be thought of as "part of" a larger category.

Below are several options about how people should feel when they live in a home that is designed or adapted to support them as they get older. Please drag and drop each one into the box you think it fits in best. You will be able to change your mind and move items between boxes as often as you like while thinking about this question.

Items

- People feel happy or content
- People feel relaxed
- People feel like their home reflects their preferences or style
- People feel sociable, or connected with others
- People feel comfortable
- People feel supported or enabled
- People feel in control of their home
- People feel valued
- People feel calm
- People feel like part of their community
- People feel warm
- People feel like their home is a private space
- People feel financially secure
- People feel like their home is a space to have fun in

Part of feeling safe and secure

Part of being able to adapt their home to suit their needs

Part of being or staying independent

Does not fit in any of the above

Section 3 of 3

One of the questions in the last survey asked about the practical benefits people should get from living in a house that is designed to support them.

We asked volunteers to choose their 'top ten' most important options from the list to help us understand their priorities, and get an insight into the kind of activities that should be supported in a home designed with ageing in mind.

This question was the most divisive among our volunteers, with only a few options gaining strong support across all groups. The graph below shows the pattern of answers we received, with the 'most important' or frequently chosen options at the top, and those less frequently chosen on the bottom.

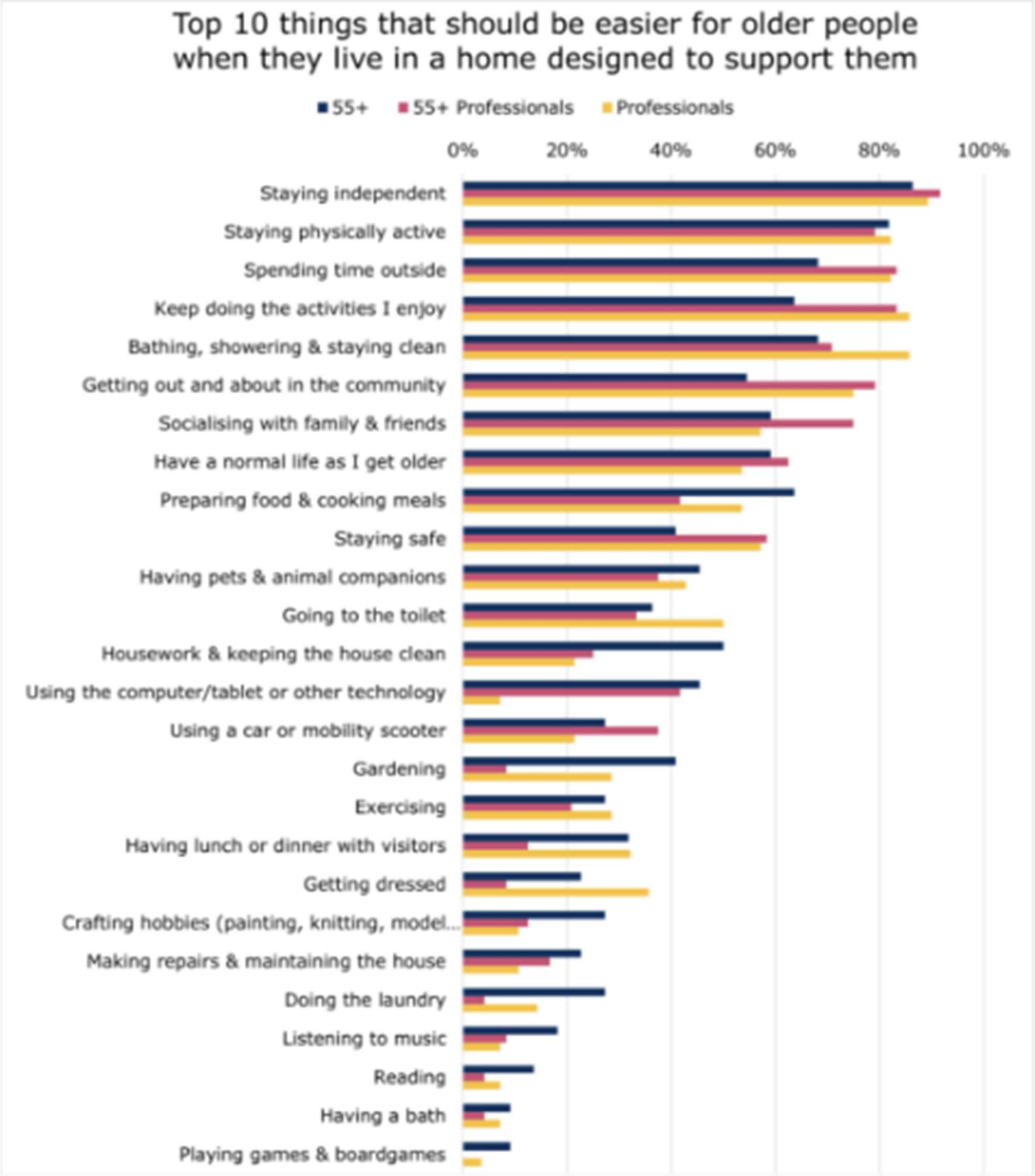

The following questions have been designed to help us understand these answers in more detail.

What do you think about the pattern of answers shown in the graph above? How well does the ranking above match your thoughts about the kind of activities that might be easier for people when they live in a home designed to support them as they age?

The next question is designed to help us understand the answers given in Round One by using the most commonly chosen options as 'sorting boxes' or umbrellas, to explore whether some of our original options might be thought of as "part of" a larger category.

Below are several options about the kinds of activities that people might find easier if they were in a home designed to support them as they age. Please drag and drop each one into the box you think it fits in best. You will be able to change your mind and move items between boxes as often as you like while thinkng about this question.

Items

Gardening

Listening to music

Reading

Getting out and about in the community

Getting dressed

Staying safe

Bathing, showering & staying clean

Playing games & boardgames

Having pets & animal companions

Using the computer/tablet or other technology

Preparing food & cooking meals

Socialising with family & friends

Having lunch or dinner with visitors

Exercising

Using a car or mobility scooter

Making repairs & maintaining the house

Crafting hobbies (painting, knitting, model building etc)

Housework & keeping the house clean

Have a normal life as I get older

Doing the laundry

Going to the toilet

Having a bath

Staying physically active

Staying independent

Keep doing activities I enjoy

None of these

How easy do you think it would be to design or adapt a home to help people keep doing the following activities as they get older?

	Extremely difficult	Somewhat difficult	Neither difficult nor easy	Somewhat easy	Extremely easy
Exercising	○	○	○	○	○
Doing the laundry	○	○	○	○	○
Having lunch or dinner with visitors	○	○	○	○	○
Making repairs & maintaining the house	○	○	○	○	○
Crafting hobbies (painting, knitting, model building etc)	○	○	○	○	○
Staying safe	○	○	○	○	○
Getting dressed	○	○	○	○	○
Using a car or mobility scooter	○	○	○	○	○
Using the computer/tablet or other technology	○	○	○	○	○
Listening to music	○	○	○	○	○
Playing games & boardgames	○	○	○	○	○
Gardening	○	○	○	○	○
Going to the toilet	○	○	○	○	○
Reading	○	○	○	○	○
Having pets & animal companions	○	○	○	○	○
Housework & keeping the house clean	○	○	○	○	○
Preparing food & cooking meals	○	○	○	○	○
Having a bath	○	○	○	○	○

End

You have now reached the end of Round 2 of the eDelphi Consultation.

We are very grateful to everyone who takes part in this process, and to those who reached out with feedback about their experience of filling in Round One of the consultation. We hope you found the format of Round 2 clearer, more engaging, and easier to move through.

If you have any feedback, comments, or suggestions for what you would like to see in the Final Round of the consulation, please enter them in the box below and click 'next' to ensure they are properly saved before closing the survey.
