# Supplementary material for: Outcomes of home design to support healthy cognitive ageing: modified e-Delphi exercise with older people and housing-related professionals
Source: BMC Geriatr. 2024 Jun 24;24:546. doi: 10.1186/s12877-024-05085-z (PMC11194886; doi:10.1186/s12877-024-05085-z)
Supplement: Supplementary file 6 — Supplementary Material 6 [file 12877_2024_5085_MOESM6_ESM.pdf]

Welcome the Final Round of the DesHCA Project's eDelphi process!

A Reminder About the Project
The Designing Homes for Healthy Cognitive Ageing (DesHCA) project is a large, UKRI funded research project based at the University of Stirling that explores the different ways that people's homes can be designed, built, or adapted to better support them as they age. Our research involves working alongside people from different walks of life, industry backgrounds, and professional settings to examine how adapting existing homes could help older people in the here and now, as we work alongside architects and builders to design more supportive homes for the future.

About this eDelphi survey
The eDelphi survey has been designed to help us learn more about what people think about when we talk about designing supportive homes, what they'd like those homes to look like, and what they'd like the creation of more supportive homes to mean- for themselves as individuals, their communities, and for the industries and professionals working to design, build, manage and adapt homes across the UK.

The questions that follow have been shaped by the answers provided by all of our volunteer panelists so far. Taking part in this round will give you an opportunity to learn about some of the insights we've collected so far, while answering new questions to help us understand key issues around designing age friendly or supportive homes.

We recommend taking this survey on a computer or tablet device due to the interactive design of some of the questions included in this round. We expect this survey to take no more than **10 - 15 minutes to complete, a**nd will close to new responses at 11.59pm on Sunday 30th of April.

What's Next?
After the survey closes, the team work to bring together all of the data collected over the three rounds of the eDelphi consultation to develop a report outlining what we've learned from the process, and how we can use this insight to advocate for the creation of more supportive homes in the future. We will email you a copy of this report, which will include summaries of all the questions we've asked thus far, as well as the answers provided during this round.

Important information

Your participation the eDelphi process remains entirely voluntary: although you have taken part in the eDelphi thus far, you do not have to continue to take part if you do not wish to, and you can withdraw from the process at any time without providing a reason.

All answers collected through the eDelphi surveys will be anonymised, meaning we will never reveal your details to those outside the study. While the survey does ask for some details about you these are only intended to provide context for the answers you give, and help us understand any patterns we notice in the data.

Information gathered by the eDelphi will be used to inform other research activities within the DesHCA project (including the design of our VR home models and our Serious Game), and may be reported in other ways such as academic papers, policy briefings, presentations, and blogs. We may use a summary of the data collected during the eDelphi surveys to do this, including picking out specific quotes or opinions to highlight key messages- but these will always be anonymised.

We hope that you feel able to complete this survey- however if at any time you wish to stop, you may do so by closing the browser window. If you exit the survey before answering the final question, your responses will not be included in the project. If you complete the survey but change your mind and wish to withdraw your responses, you may do so by emailing Catherine Pemble at catherine.pemble@stir.ac.uk within 2 weeks of completing the survey and providing your name.

If you experience problems accessing this survey online and would like to complete the survey over the telephone instead, then please email catherine.pemble@stir.ac.uk to arrange a time to do this. If you would like further information about this project, you can email the Principal Investigator, Professor Alison Bowes, at a.m.bowes@stir.ac.uk. If you have concerns about this survey, or the conduct of this project, them please contact Professor Liz Forbat, Deputy Dean of the Faculty of Social Sciences, by email at elizabeth.forbat1@stir.ac.uk.

If you understand the information above, and are happy to complete the next round of the eDelphi click 'Next' to proceed to the next screen.

Please confirm your details below. We will use this information to match your answers with your responses to the previous rounds of the eDelphi survey.

What is your name?

Please re-enter your email address below.

Feedback From Round 2

1 of 2

Several of our questions in Round 2 were designed to help us understand why some activities, or experiences, might have been ranked as part of people's 'top ten' when others did not. Many of our volunteers in Round 2 suggested that this might be a result of people choosing 'overarching' options, such as 'Staying Independent' as one of their top ten over more specific options.

We explored this idea by having volunteers in Round 2 drag-and-drop activities into different categories. The infographic below shows the outcome of this activity, with votes for "Staying Independent" shown in yellow, votes for "Physical Activity" shown in blue, votes for "Enjoyed Activities" shown in green, and the remainder votes shown in grey.

This means that, for example, 10% of people catagorised Gardening as part of Staying Independent, 59% thought it was part of Physical Activity, 29% said it was part of an Enjoyed Activity, with 2% of volunteers feeling that it did not fit within any of these categories.

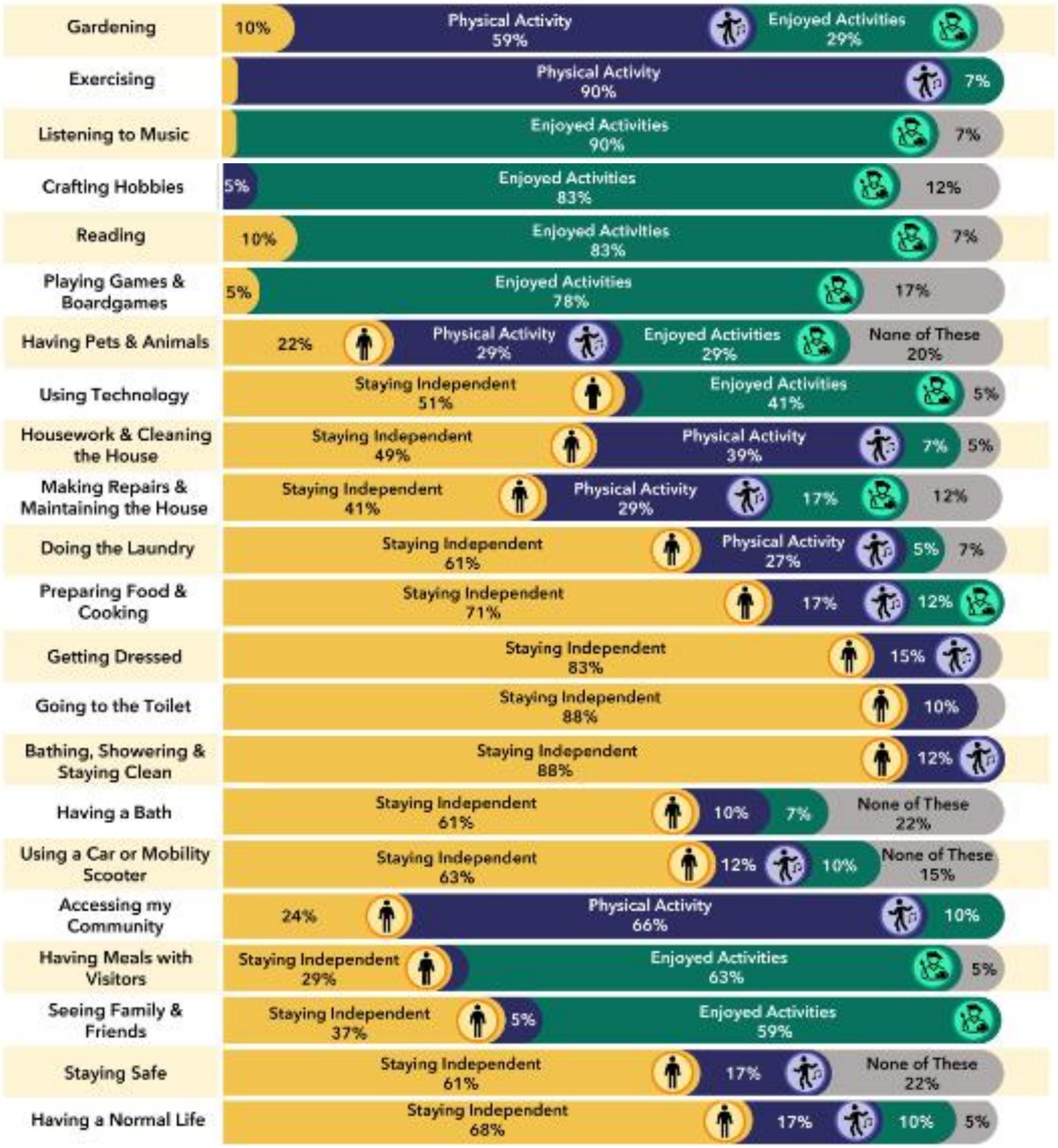

What do you think about the pattern of answers shown above? Are there any areas of strong agreement, or disagreement, that surprise you?

Feedback From Round 2

During the last round we asked volunteers to arrange the different feelings, or experiences people should have as a result of living in a more supportive home in the same way.

Using the results from Round 1 we created three overarching categoriesof 'feeling' for people to sort the options into. These categorieswere Being or Staying Independent (shown in yellow), Feeling Safe and Secure (shown in green), and Able to Adapt their Home (shown in purple), as well as the 'None of These' option (shown in grey).

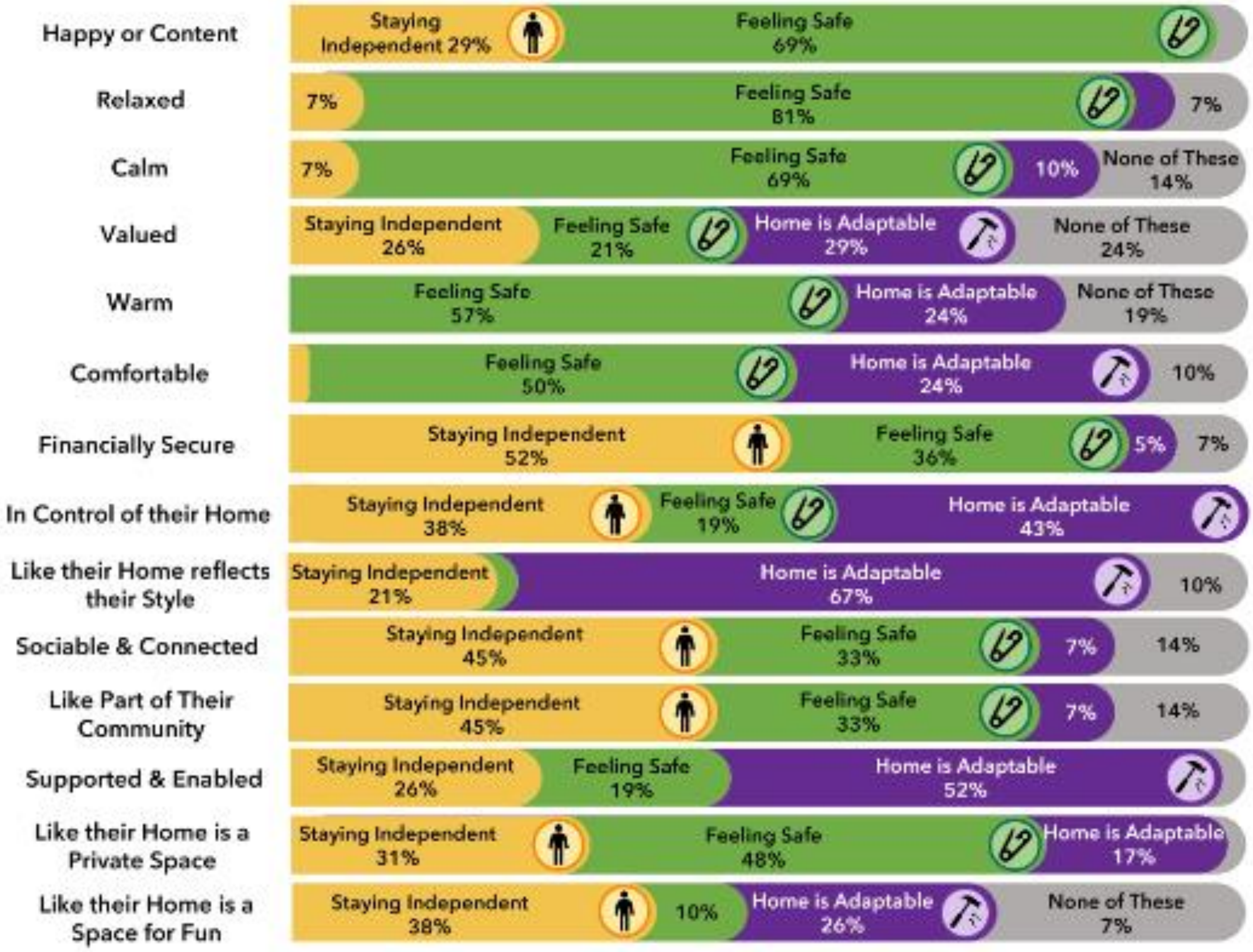

What do you think about the pattern of answers shown above? Are there any areas of strong agreement, or disagreement, that surprise you?

Questions for Round 3

We want to use Round 3 of this eDelphi Consultation to learn more about the way that people think building more supportive or age friendly homes will impact different industries within the UK.

Many of these questions build on insights we gained in Round 1, while giving us an opportunity to dig deeper into how our different groups of volunteers think about the future of supportive home design.

We hope that these answers will help us to understand the different hopes and fears people have around supportive design, as well as helping to identify key challenges and roadblocks that might make it difficult to change the way we think about home design in the UK.

We want to learn more about how you think taking steps to build homes that support people as they age might positively or negatively impact different businesses and industries within the UK.

Please read the statements below and select the options that best represent you opinion.

For example, if you think that changing government legislation to support the creation of more age friendly homes would have a positive impact on professionals working in housing design, but a negative impact for professionals working in construction, you would select 'positive' in the first column, and 'negative' in the second etc.

	For Professionals in Home & Housing Design	For Professionals in Building & Construction	For Professionals in Sales & Housing Supply	For Professionals in Housing Management	For Professionals in Health & Social Care
I think the result of changing government legislation to drive the creation of more supportive homes would be...					
I think the result of changing industry rules and regulations to require homes to be more supportive would be...					
I think the result of raising awareness of supportive design and age friendly homes within the industry would be...					
I think the result of educating home owners and renters about the principles of supportive design and age-friendly homes would be...					

Volunteers in the first round of the eDelphi consultation were asked to choose the 'top ten' ways they thought building more supportive homes might benefit different industries

into these answers from the perspectives of our three groups of volunteers- people over 55, industry professionals who are over 55, and industry professionals who are under 55.

Please reach each of the statements carefully, and select the options that best represent your opinion (you can choose as many or as few as you like).

Each question also gives you the opportunity to select "It's complicated and I would like to say more", which will create a text box on the following page where you can give more information about your thoughts and insights.

Please read the statement below, and tick the options that you most agree with. You can select as many or as few as you like.

Statement 1 of 5:
I think that companies and organisations who are seen or known to create more supportive or age-friendly homes would have a better reputation than those that do not.

- ☐ I think this is true for companies and organisations who design homes (architects, designers etc.)
- ☐ I think this is true for companies and organisations who build homes (builders, construction companies etc.)
- ☐ I think this is true for companies and organisations who supply homes (house builders, housing associations, and local authorities etc.)
- ☐ I think this is true for companies and organisations who manage homes (local authorities, housing associations etc.)
- ☐ It's complicated and I would like to say more

You selected the "It's complicated and I would like to say more" option for statement 1 of 5, "I think that companies and organisations who are seen or known to create more supportive or age-friendly homes would have a better reputation than those that do not."

You selected the "It's complicated and I would like to say more" option for statement 1 of 5, "I think that companies and organisations who are seen or known to create more supportive or age-friendly homes would have a better reputation than those that do not."

Please use the box below to add your insights or comments.

Please read the statement below, and tick the options that you most agree with. You can select as many or as few as you like.

Statement 2 of 5:
I think that companies and organisations who are seen or known to create more supportive or age-friendly homes would win more contracts or attract more business than those that do not.

- ☐ I think this is true for companies and organisations who design homes (architects, designers etc.)
- ☐ I think this is true for companies and organisations who build homes (builders, construction companies etc.)
- ☐ I think this is true for companies and organisations who supply homes (house builders, housing associations, and local authorities etc.)
- ☐ I think this is true for companies and organisations who manage homes (local authorities, housing associations etc.)
- ☐ It's complicated and I would like to say more

You selected the "It's complicated and I would like to say more" option for statement 2 of 5, "I think that companies and organisations who are seen or known to create more supportive or age-friendly homes would win more contracts or attract more business than those that do not."

Please use the box below to add your insights or comments.

Please read the statement below, and tick the options that you most agree with. You can select as many or as few as you like.

Statement 3 of 5:
I think that companies and organisations who are seen or known to create more supportive or age-friendly houses will have access to customers or markets that other businesses do not.

- ☐ I think this is true for companies and organisations who design homes (architects, designers etc.)
- ☐ I think this is true for companies and organisations who build homes (builders, construction companies etc.)
- ☐ I think this is true for companies and organisations who supply homes (house builders, housing associations, and local authorities etc.)
- ☐ I think this is true for companies and organisations who manage homes (local authorities, housing associations etc.)
- ☐ It's complicated and I would like to say more

You selected the "It's complicated and I would like to say more" option for statement 3 of 5, "I think that companies and organisations who are seen or known to create more supportive or age-friendly houses will have access to customers or markets that other businesses do not."

Please use the box below to add your insights or comments.

Please read the statement below, and tick the options that you most agree with. You can select as many or as few as you like.

Statement 4 of 5:
I think that creating more supportive or age-friendly homes would create more job opportunities for professionals in the UK

- ☐ I think this is true for companies and organisations who design homes (architects, designers etc.)
- ☐ I think this is true for companies and organisations who build homes (builders, construction companies etc.)
- ☐ I think this is true for companies and organisations who supply homes (house builders, housing associations, and local authorities etc.)
- ☐ I think this is true for companies and organisations who manage homes (local authorities, housing associations etc.)
- ☐ It's complicated and I would like to say more

You selected the "It's complicated and I would like to say more" option for statement 4 of 5, "I think that creating more supportive or age-friendly homes would create more job opportunities for professionals in the UK"

Please use the box below to add your insights or comments.

Please read the statement below, and tick the options that you most agree with. You can select as many or as few as you like.

Statement 5 of 5:
I think that creating more supportive or age-friendly homes would help professionals feel more job satisfaction.

- ☐ I think this is true for companies and organisations who design homes (architects, designers etc.)
- ☐ I think this is true for companies and organisations who build homes (builders, construction companies etc.)
- ☐ I think this is true for companies and organisations who supply homes (house builders, housing associations, and local authorities etc.)
- ☐ I think this is true for companies and organisations who manage homes (local authorities, housing associations etc.)
- ☐ It's complicated and I would like to say more

You selected the "It's complicated and I would like to say more" option for statement 5 of 5, "I think that creating more supportive or age-friendly homes would help professionals feel more job satisfaction."

Please use the box below to add your insights or comments.

Insights From Round 1

1 of 1

The questions in this round were created to help us learn more about the answers we received in response to asking people to chose the 'top ten' ways they thought building more supportive or age friendly homes could benefit companies and organisations in the UK.

We wanted to use this final round of the eDelphi to learn more about why different groups might have chosen the options they did- and why the answers given by people over 55 might look different from those given by younger professionals working in the fields of building design, construction, supply or management.

We noticed that professionals under the age of 55 were less likely to choose options about new job opportunities, new contracts, or new opportunities for profit among their 'top ten' benefits for building more supportive homes than those in other groups. They were also less likely to choose positive PR or reputation as a major benefit of building more supportive homes. This pattern was repeated across different areas of industry, as you can see on the four charts below:

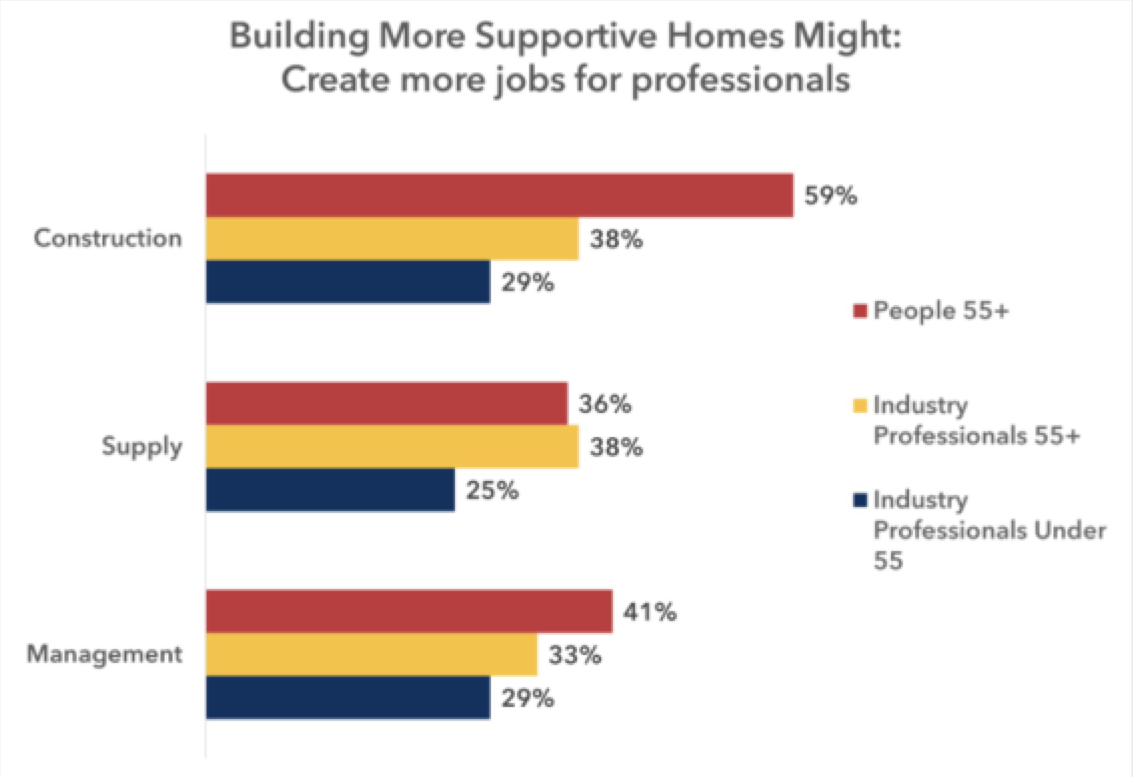

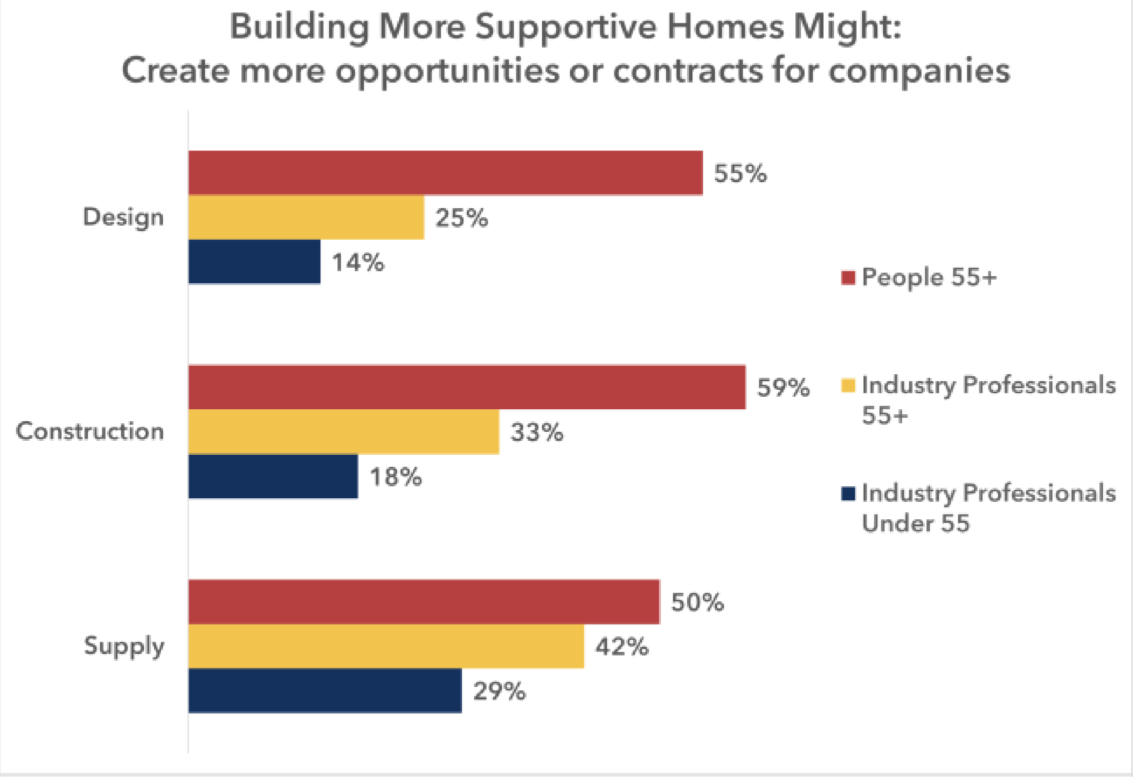

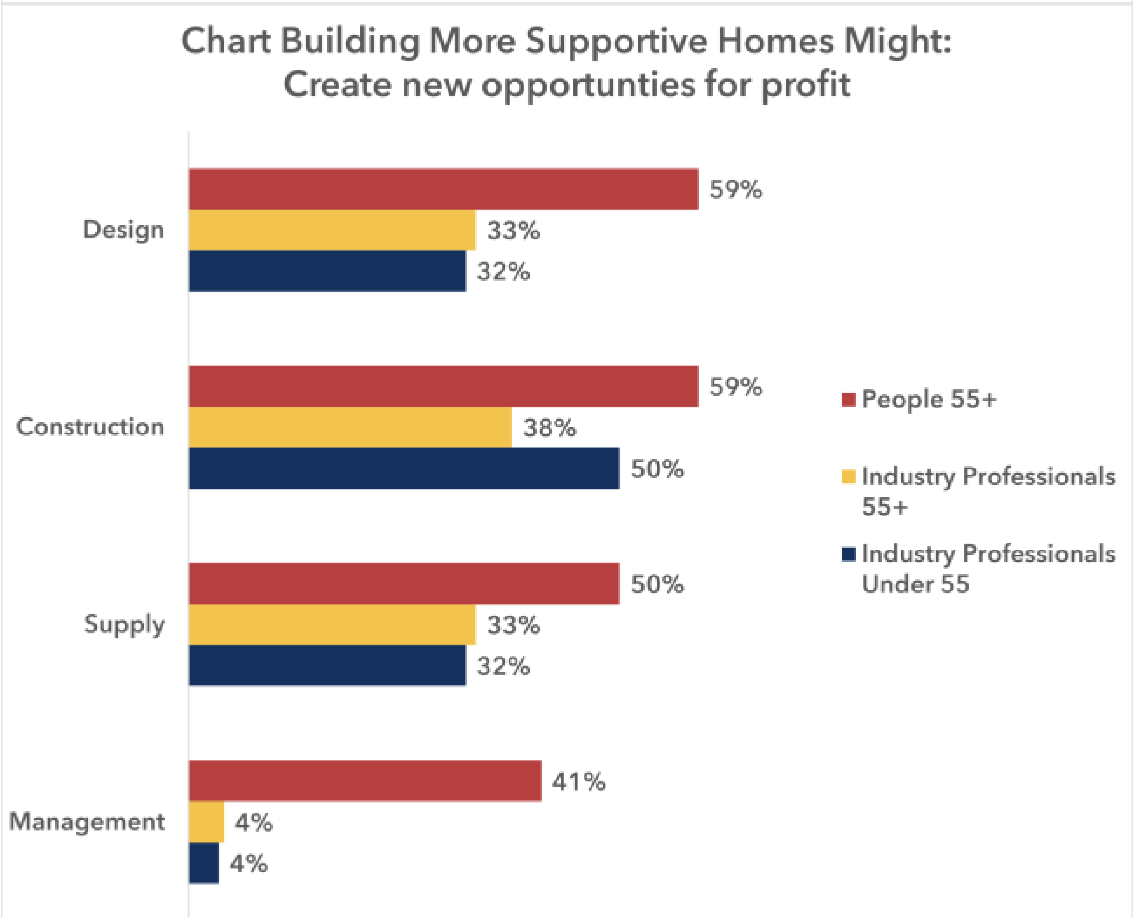
Chart Building More Supportive Homes Might:
Create new opportunties for profit
Design
59%
33%
32%
Construction
59%
38%
50%
Supply
50%
33%
32%
Management
41%
4%
4%
People 55+
Industry Professionals 55+
Industry Professionals Under 55

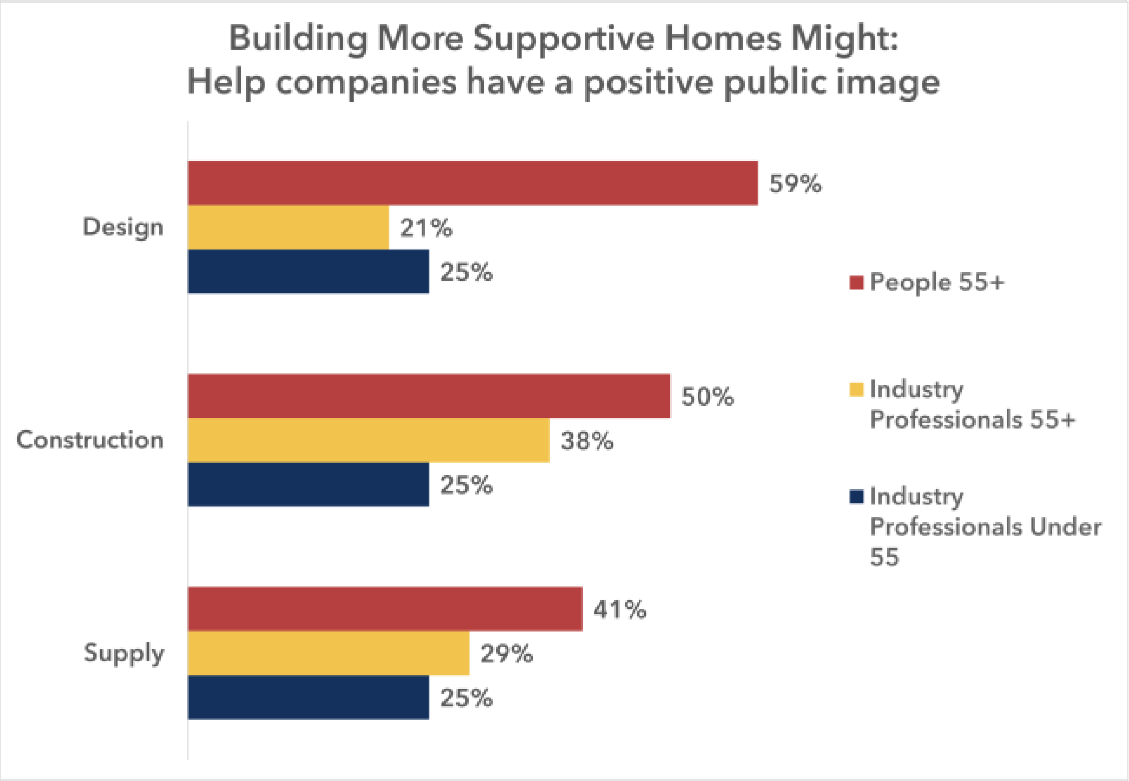
Building More Supportive Homes Might:
Help companies have a positive public image
Design
59%
21%
25%
Construction
50%
38%
25%
Supply
41%
29%
25%
People 55+
Industry Professionals 55+
Industry Professionals Under 55

What do you think about the patterns shown above? What do you think might explain these differences?

Is there anything you can think of that might change these perspectives?

DesHCA
DESIGNING HOMES FOR HEALTHY COGNITIVE AGEING

Thank you for taking part in the Designing Homes for Healthy Cognitive Ageing projects eDelphi Consultation- we are very grateful for your time, and all of the insight you've provided to the project throughout this process.

These final four questions are designed to help us understand and learn from your experience as a volunteer panelist in the eDelphi Consultation.

Is there anything in particular we should *stop*, or try to avoid if we use this method again?

Is there anything in particular we should *start*, or try doing if we use this method again?

Is there anything in particular we should *continue*, or do in the same way if we use this method again?

Is there any other advice or feedback that you would like to give to the research team?

You have now reached the end of the eDelphi Consultation.

We are very grateful to everyone who takes part in this process, and to those who reached out with feedback about their experience of taking part in the consultation.

You will not receive any more eDelphi survey invitations from the DesHCA team, but you will receive an email that includes a short report explaining the findings of this process once the research team has analysed all of the data collected.

If you would like to stay in contact with the DesHCA project, or hear more about the different ways you can take part in our research, you can visit our website at www.deshca.co.uk, or email the team at DesHCAResearch@stir.ac.uk.
